# Gender differences in related influential factors of regular exercise behavior among people in Taiwan in 2007: A cross-sectional study

**DOI:** 10.1371/journal.pone.0228191

**Published:** 2020-01-31

**Authors:** Hsin-Yu Mao, Hui-Chuan Hsu, Shin-Da Lee

**Affiliations:** 1 Department of Health Care Administration, Asia University, Taichung, Taiwan; 2 Department of Physical Therapy, Tzu-Hui Institute of Technology, Pingtung, Taiwan; 3 School of Public Health, Taipei Medical University, Taipei, Taiwan; 4 Department of Occupational Therapy, Asia University, Taichung, Taiwan; 5 Department of Physical Therapy, China Medical University, Taichung, Taiwan; 6 School of Rehabilitation Science, Shandong University of Traditional Chinese Medicine, Shandong, China; Teesside University/Qatar Metabolic Institute, UNITED KINGDOM

## Abstract

**Purpose:**

The purpose was to explore the exercise behavior of the Taiwanese population during leisure time and to examine the gender differences in related influential factors.

**Methods:**

The Leisure Time and Sport Questionnaire (LTSQ) conducted by the Academia Sinica in 2007 was used for analysis. Participants were drawn from the Taiwanese population aged over 18 years old. Multinomial logistic regression models were used to test the association between the frequency of exercise and related factors in all the participants, male, and female participants, respectively.

**Results:**

In the total 2,147 participants, 47.8% reported they regularly exercised, 34.1% irregularly exercised, and 18.1% never exercised. There were no significant gender differences in the frequency of exercise, but a significant gender difference was found in the types of exercise most often practiced. Participants in one of following conditions, over 65 and under 40 years old, living in the city, having higher education levels, having a spouse or partner, enjoying exercise, feeling worthwhile to spend money on exercise, and not having to take care of others, were more likely to be engaged in regular exercise in the total population. The “enjoyment” in exercise was a significant influential factor for both sexes. Women were more likely to exercise regularly if they were aged 40–64 years old compared with those over 65 years old, have lower self-rated health scores, felt it was more worthwhile to spend money on exercise and did not have to care for another, whereas men who have higher education level and did not feel a lack of time were more likely to exercise regularly.

**Conclusions:**

There are gender differences in regular exercise behavior during leisure time and related influential factors among Taiwanese adults. The findings of the present study can be used when designing gender-specific health promotion programs.

## 1. Introduction

The benefits of being physically active have been well documented for all age groups including controlling body weight, promoting musculoskeletal functions, enhancing cardiovascular fitness, reducing the risks of coronary heart diseases, stroke, diabetes, colon cancer, breast cancer and falls [1–3]. Regularly engaging in physical activities can improve psychological statuses such as increasing self-confidence, promoting social interaction, and preventing depression [4, 5, 6]. The American College of Sports Medicine recommends that adults should perform either moderate aerobic exercise for a minimum of 30 minutes five days a week, or vigorous exercise for a minimum of 20 minutes three days a week, or an equivalent combination of moderate- and vigorous-intensity activities to gain health benefits [3]. However, in an international prevalence study, 42.3% of the Taiwanese population was in the low activity category defined by the International Physical Activity Questionnaire (IPAQ), which means that people engage in physical activities less than three days a week and in a mild intensity [7]. Another study by Wen et al. (2007) compared the intensity of leisure time physical activity between Taiwan and United States. The authors found that the prevalence of leisure time physical activity is lower in the Taiwanese population generally. Moreover, they found that the prevalence decreased with age in the U.S., but peaked with the elders (65 years of age or older) in Taiwan [8]. The most serious deficiency was found among the young adults (25–44 years of age).

Understanding the determinants that influences the behavior of being physically active is important for developing interventions of regular exercise. Currently known positive influential factors of physical activity include male gender, younger age, higher education level, being married, positive attitudes and social support [1, 9, 7, 10, 11, 12, 13, 14, 15, 16]. Self-efficacy, enjoyment gained in physical activities, and social support have been found to be significant correlates of physical activity in Singapore adolescents [15]. Self-efficacy is defined as belief in one’s ability to be physically active, meet goals, and overcome barriers [1, 17–20, 21]. Social support can be attained from family or friends [1, 9, 12–15, 20]. The likelihood of meeting the recommendations for physical activity in American adults at least doubled when people have company to exercise with [20].

Barriers to exercise include environmental barriers, cost considerations, poor health condition, fear of injury, and lack of time [17, 18, 19, 20, 22]. Individuals who perceive more barriers to being physically active are less likely to have a regular exercising behavior [1, 17–20, 23]. Child care needs and health problems have been found to be common barriers to regular exercise for low income women [17]. Lack of time and potential for injury are the primary barriers to regular exercise in older adult populations [1]. Having no access to exercising facilities or environments (such as parks) were also considered a barrier [19].

Several studies have noted gender differences in exercise participation [5, 24, 25]. Men usually participate more in regular exercise than women [7]. Gender roles may affect regular exercise behavior in traditional oriental cultures, in which women mostly play the role of caregivers in the family and have less time for exercise [14, 26]. Only two studies have explored the gender difference of motivation to exercise in Asian countries. One study in Malaysia indicated that the motivations to exercise for men were more related to intrinsic factors, such as gaining strength, competition, and challenge. In contrast, women’s motivations were more related to extrinsic factors, such as weight management and attaining an attractive appearance [25]. The other study in Taiwan suggested that men’s intention to exercise was to improve their appearance and because of their personal interest in sports. On the other hand, women were more motivated to do exercise when Taiwanese athletes performed well in international sporting competitions as a result of the celebrity effect [5].

Few studies have analyzed the determinants of exercise behavior in Asian countries. However, self-rated health, self-perceived happiness, perceptions of exercise, having company to exercise, and barriers to exercise have not been analyzed in previous studies. Therefore, the purpose of this study was to attain a better understanding of the exercise behavior in an Asian population and to examine the influential factors of regular exercise behavior. We also examined gender differences in the influential factors that enhanced or hampered regular exercise in order to provide implications for future health policies and health promotion practices.

## 2. Materials and methods

### 2.1 Data and participants

The original data were from the 2007 Taiwan Social Change Survey (TSCS) conducted by the Academia Sinica, Taiwan. The TSCS survey is collected every year on different topics, and the same topic is repeatedly collected every 5 years. The 2007 TSCS data included a supplemental module, the Leisure Time and Sport Questionnaire (LTSQ), in which the participants were drawn from the Taiwanese population aged 18 years or older by a three-stage stratified probability sampling method that used Taiwan census data as the sampling frame. In total 2,147 people completed the LTSQ survey, which was used for the analysis in this study. This study used secondary data for analysis. All the participants were introduced to the study’s purpose, explained and guaranteed the protection of their anonymity, and all of them were voluntary. The study was approved by the Central regional research ethics committee, China Medical University, Taiwan (Approval No. CRREC-108-107).

### 2.2 Measures

#### 2.2.1 Dependent variable

The dependent variable of this study was the frequency of exercise. Exercise was defined as physical activities performed during leisure time that were planned, structured, and repetitive for the purpose of conditioning the body. “Leisure time”referred to the free time outside of work, housework and other daily activities that had to be done regularly and could be arranged by one’s own will. As a result,“exercise”in the present study was not confused with activities completed during work, transportation to work or housework [16]. We used the question, “How often do you engage in physical activities in your leisure time (e.g., exercising, going to the gym or walking)?” to examine the frequency of exercise. We then categorized the 5-point Likert scale responses and recoded “every day” and “several times a week” as regular exercise, “several times a month” and “several times a year or less” as irregular exercise, and “never” as no exercise.

#### 2.2.2 Control variables

The demographic factors included age, gender, living area, marital status, working status, and educational level. All participants were categorized into three age groups: <40, 40–64, or ≧65 years old. Living areas were categorized as countryside, suburbs, or city. Marital statuses were divided into having or not having a spouse or partner. Working statuses were divided into having or not having a full-time job. Participants’ education levels were categorized into five groups: illiterate, elementary school, high school, college, or master’s/doctoral degree or above. If the Participants were illiterate, they were assisted by the interviewer to fill out the questionnaire.

#### 2.2.3 Self-rated health

Self-rated health was scored on a scale from 1 to 5 indicating “poor” to “excellent” health. (poor, fair, good, very good, & excellent).

#### 2.2.4 Self-perceived happiness

Happiness was assessed by asking the participants “Overall, how happy would you say you are?”. The responses ranged from 1 to 4, indicating “not happy at all” to “very happy.” (not happy at all, not so happy, happy, & very happy).

#### 2.2.5 Perceptions of exercise

The perception of exercise variables included “Enjoyment” and “Worth spending money on exercise”. “Enjoyment” was assessed by asking the participants “How much enjoyment do you gain when you are engaged in physical activities in your leisure time (e.g., exercising, going to the gym or walking)?”. The responses were on a scale from 1 to 5, indicating “do not enjoy at all” to “a lot of enjoyment.” (do not enjoy at all, not so much enjoyment, some enjoyment, fairly enjoy, & a lot of enjoyment) The item used to measure “Worth spending money on exercise” was “Do you feel that it is worthwhile to spend money on exercise?”. The answers ranged from 1 to 4, indicating “not worthwhile at all” to “very worthwhile.” (not worthwhile at all, not so worthwhile, fairly worthwhile, & very worthwhile).

#### 2.2.6 Social support

Social support was surveyed by asking the participants if they had company with whom they exercised. “Have company when exercising” was assessed by asking “Do you usually exercise by yourself or with company (family or friends)?”. The participants answered on a 5-point Likert scale from 1 to 5, indicating “always alone” to “always with company.” (always alone, mostly alone, 50% with company, mostly with company, & always with company).

#### 2.2.7 Barriers to exercise

The participants answered the question “How much would the following factors influence the frequency of your exercise during leisure time?” for five barriers including lack of facilities nearby, lack of money, poor personal health condition (e.g., age, disability, etc.), care of others (elderly or children), and lack of time. The responses ranged from 1 to 4, indicating “no influence at all” to “very strong influence.” Higher scores indicated that the barrier had a greater influence on the frequency of exercise.

#### 2.2.8 Types of exercise

The participants were asked to provide the exercises they did most often. We categorized all of the exercises reported into five groups: Competitive sports, Strengthening exercises, Aerobic exercises, Walking, or Recreational activities. Competitive sports referred to team sports (e.g., basketball, football, etc.) or racket sports (e.g., badminton, tennis, etc.) in which players would have to compete with another team or opponents. Strengthening exercises included resistance training executed in a gym or other places. Aerobic exercises included jogging, aerobic dancing, swimming, or other exercises that emphasized on using large muscle groups and cardiopulmonary training. Walking is an exercise of mild intensity and could be performed almost in any location. Recreational activities included Chi-Gong, stretching exercises, yoga, and other physical activities with low intensity or that were solely for recreation (e.g., fishing, gardening, etc).

### 2.3 Statistical analysis

All statistical analysis was performed using the Statistical Package for Social Science software version 20.0 (SPSS, IBM Corp., Armonk, New York). A descriptive analysis and bivariate analysis by gender were first conducted using chi-square and independent t-tests. The gender difference in exercise types in the regular and irregular exercise groups (no exercise group excluded) were analyzed by chi-square test, respectively. Finally, multinomial logistic regression models were used to test for associations between the frequency of exercise and related influential factors. The dependent variables were categorized into three groups: no exercise, irregular exercise, or regular exercise. The no exercise group was the reference group. All data of the participants were included in the multinomial logistic regression model (Model 1), and the participants were then divided by gender (Model 2 for men and Model 3 for women).

## 3. Results

The descriptive data of the total participants and the characteristics by gender are in Table 1. The total participants were composed of 50.2% male and 49.8% female. Of the total participants, 39.8% were 18–39 years old, 43.8% were 40–64 years old, and 16.3% were 65 years or older. Of all the participants, 47.8% regularly exercised, 34.1% irregularly exercised, and 18.1% never exercised. There were no significant differences in exercise frequency by gender.

**Table 1:** Characteristics of the study participants (% or mean (SD)).

Variables	Total (n = 2147)	Male (n = 1078)	Female (n = 1069)	Significance
**Gender**	100%	50.2%	49.8%	
**Age (years old)**				NS
18–39	39.8%	41.1%	38.5%	
40–64	43.8%	42.5%	45.2%	
≧65	16.3%	16.4%	16.3%	
**Living environment**				NS
City	21.8%	21.9%	21.8%	
Suburbs	29.6%	30.5%	28.6%	
Countryside	48.6%	47.6%	49.6%	
**Level of education**				***
Uneducated	7.0%	2.6%	11.5%	
Elementary school	19.0%	17.3%	20.8%	
High school	39.7%	43.9%	35.5%	
College	27.7%	28.5%	26.8%	
Master’s/Doctoral degree	6.5%	7.7%	5.3%	
**Marital status**				NS
Have a spouse or partner	64.8%	66.8%	62.8%	
No spouse or partner	35.2%	33.2%	37.2%	
**Working status**				***
Have full-time work	62.3%	68.9%	55.7%	
Part-time work/ Student/ Housewife	37.7%	31.1%	44.3%	
**Frequency of exercise**				NS
Regular exercise	47.8%	49.9%	45.7%	
Irregular exercise	34.1%	33.1%	35.1%	
No exercise	18.1%	17.0%	19.2%	
**Self-rated health**	3.06 (0.61)	2.98 (1.13)	2.73 (1.13)	***
**Self-perceived happiness**	2.86 (1.14)	3.07 (0.61)	3.06 (0.60)	NS
**Perceptions of exercise**				
Enjoyment	3.56 (0.92)	3.62 (0.89)	3.50 (0.96)	**
Worth spending money on exercise	2.54 (0.86)	2.60 (0.86)	2.49 (0.85)	**
**Social support**				NS
Have company when exercising	3.41 (1.22)	3.41 (1.23)	3.40 (1.26)	
**Barriers to exercise**				
Lack of facilities	2.20 (1.10)	2.21 (1.10)	2.20 (1.10)	NS
Lack of money	2.23 (1.12)	2.18 (1.11)	2.28 (1.12)	*
Poor health status	2.21 (1.12)	2.16 (1.09)	2.25 (1.12)	NS
Taking care of children or the elderly	1.84 (1.00)	1.74 (0.92)	1.95 (1.04)	***
Lack of time for exercise	2.11 (1.04)	2.03 (1.03)	2.20 (1.05)	***

Gender difference was analyzed by Chi-square and independent t-test.

*p <0.05,

**p <0.01,

***p <0.001. NS: non-significant

A significant gender difference was found in the level of education, working status, self-rated health, and the perceptions of exercise. A larger proportion of men received higher education, have full time work, and have higher scores in self-rated health (mean = 2.98, SD = 1.33). The enjoyment gained from exercise was greater in men (mean = 3.62, SD = 0.89) than in women (mean = 3.50, SD = 0.96). Men felt that it was more worthy to spend money on exercise (mean = 2.60, SD = 0.86) than women (mean = 2.49, SD = 0.85). However, there was no significant gender difference in living environment, marital status, self-perceived happiness, and having company to exercise with or not.

Of the five barriers to exercise, lack of money was the largest barrier (mean = 2.23, SD = 1.12), and taking care of children/elderly was the smallest (mean = 1.84, SD = 1.00) for the total population. There were significant gender differences in the barriers to exercise: lack of money, taking care of children/elderly, and lack of time. All the three barriers were larger barriers for women than men (p<0.000).

The most frequent type of exercise performed by the responders are shown in Table 2. Only those who reported that they irregularly or regularly exercised were included and analyzed separately. There was a significant difference between the types of exercise performed by men and women (p<0.001). The percentages of male participants who regularly exercised most often involved in competitive sports, strengthening exercises, aerobic exercises, walking, and recreational activities were 20.2%, 3.4%, 34.7%, 34.7%, and 7.0%, respectively; on the other hand, the percentages of female participants were 5.0%, 0.84%, 17.4%, 54.1%, and 22.6%, respectively. Similar trends of percentages also were found for irregular exercising male and female responders.

**Table 2:** Type of exercise most often performed by individuals who regularly or irregularly exercise (n = 1626).

	Total		Male ***		Female***	
Exercise type	Irregular exercise (n = 619)	Regular exercise (n = 1007)	Irregular exercise (n = 304)	Regular exercise (n = 530)	Irregular exercise (n = 315)	Regular exercise (n = 477)
**Competitive sports**	111 (17.9%)	131 (13.0%)	85 (28.0%)	107 (20.2%)	26 (8.3%)	24 (5.0%)
**Strengthening exercise**	10 (1.6%)	22 (2.2%)	6 (2.0%)	18 (3.4%)	4 (1.3%)	4 (0.84%)
**Aerobic exercise**	195 (31.5%)	267 (26.5%)	105 (34.5%)	184 (34.7%)	90 (28.6%)	83 (17.4%)
**Walking**	240 (38.8%)	442 (43.9%)	83 (27.3%)	184 (34.7%)	157 (49.8%)	258 (54.1%)
**Recreational activities**	63 (10.2%)	145 (14.4%)	25 (8.2%)	37 (7.0%)	38 (12.1%)	108 (22.6%)

Gender difference in exercise type was analyzed by Chi-square test in the irregular and regular exercise group, respectively.

***p <0.001.

The factors related to the frequency of exercise and the odds ratios are in Table 3. In Model 1, the factors associated with the frequency of exercise included: (1) age group; (2) living area; (3) marital status; (4) level of education; (5) pleasure in exercise; (6) feelings about the worth of spending money on exercise; and (7) the barrier of having to take care of others. Participants aged 40–64 years were less likely to exercise regularly than those aged over 65 years (OR = 0.39, p = 0.017). Participants living in the countryside or the suburbs were less likely to exercise regularly or irregularly than those living in the city (ORs ranged from 0.45–0.49; p values ranged from 0.028–0.045). Moreover, those who did not have a spouse or partner were also less likely to exercise regularly (OR = 0.58; p = 0.045). Participants with a higher education level were more likely to exercise regularly (OR = 1.56, p = 0.007) or irregularly (OR = 1.66, p = 0.002) than to never exercise. There was a strong relationship between more enjoyment in exercise and better exercising behavior (OR = 2.32 for irregular exercise and OR = 4.60 for regular exercise; p<0.001). There was also a strong relationship between feeling that it was more worthwhile to spend money on exercise and better exercising behavior (ORs were 1.51 and 1.56 for irregular and regular exercise, respectively; p = 0.002). However, gender and have company when exercising were not significant factors. Regarding the barriers to exercise, participants who felt more influenced by the barrier of “having to take care of others” were less likely to exercise regularly or irregularly (OR = 0.68 for irregular exercise, p = 0.003 and OR = 0.72 for regular exercise, p = 0.010). The remaining barriers (lack of facilities, lack of money, poor health, and lack of time) were not significant for the total participants.

**Table 3:** Factors related to regular or irregular exercise behavior (irregular exercise vs. no exercise; regular exercise vs. no exercise).

	Model 1: Total				Model 2: Male				Model 3: Female			
	Irregular exercise		Regular exercise		Irregular exercise		Regular exercise		Irregular exercise		Regular exercise	
	OR (95%CI)	*Sig*.	OR (95%CI)	*Sig*.	OR (95%CI)	*Sig*.	OR (95%CI)	*Sig*.	OR (95%CI)	*Sig*.	OR (95%CI)	*Sig*.
**Gender (reference: Female)**												
Male	0.84 (0.53–1.34)		0.93 (0.58–1.49)									
**Age (years) (reference: ≧65 years)**												
18–39	2.35 (0.87–6.36)		0.48 (0.18–1.26)		2.34 (0.53–10.40)		0.58 (0.13–2.51)		3.36 (0.82–13.79)		0.46 (0.12–1.78)	
40–64	0.92 (0.40–2.12)		0.39 (0.18–0.84)	*	1.26 (0.37–4.36)		0.63 (0.19–2.08)		0.92 (0.29–2.92)		0.29 (0.10–0.85)	*
**Living environment (reference: City)**												
Countryside	0.49 (0.25–0.98)	*	0.49 (0.25–0.97)	*	0.42 (0.13–1.37)		0.46 (0.14–1.50)		0.57 (0.24–1.36)		0.51 (0.21–1.22)	
Suburbs	0.45 (0.22–0.92)	*	0.48 (0.23–0.99)	*	0.29 (0.09–0.95)	*	0.35 (0.11–1.16)		0.65 (0.25–1.68)		0.58 (0.23–1.50)	
**Level of education**	1.66 (1.20–2.29)	**	1.56 (1.13–2.15)	**	2.17 (1.23–3.84)	**	2.47 (1.40–4.38)	**	1.35 (0.88–2.05)		1.08 (0.71–1.64)	
**Marital status (reference: Have a spouse or partner)**												
No spouse or partner	0.64 (0.37–1.08)		0.58 (0.34–0.99)	*	0.66 (0.28–1.61)		0.76 (0.31–1.87)		0.68 (0.34–1.35)		0.47 (0.24–0.94)	*
**Working status (reference: Full-time work)**												
Part-time work/ Student/House wife	1.01 (0.60–1.70)		1.39 (0.82–2.34)		0.98 (0.40–2.41)		1.66 (0.68–4.08)		1.11 (0.56–2.19)		1.17 (0.60–2.31)	
**Self-rated health**	0.82 (0.66–1.02)		0.87 (0.70–1.09)		0.92 (0.66–1.28)		0.96 (0.69–1.34)		0.74 (0.54–0.99)	*	0.80 (0.59–1.07)	
**Self-perceived happiness**	1.12 (0.76–1.66)		1.15 (0.78–1.70)		1.20 (0.67–2.16)		1.42 (0.79–2.57)		1.11 (0.65–1.91)		0.97 (0.57–1.65)	
**Perception of exercise**												
Enjoyment	2.32 (1.77–3.04)	***	4.60 (3.48–6.08)	***	3.39 (2.16–5.32)	***	6.89 (4.32–10.99)	***	1.82 (1.26–2.62)	*	3.60 (2.47–5.16)	***
Worth spending money	1.51 (1.15–1.99)	**	1.56 (1.18–2.05)	**	1.49 (0.98–2.26)		1.56 (1.02–2.37)		1.46 (1.00–2.14)	*	1.49 (1.03–2.17)	*
**Social support (reference: Have no company when exercising)**												
Have company when exercising	1.10 (0.91–1.32)		0.99 (0.82–1.19)		0.92 (0.68–1.23)		0.88 (0.66–1.82)		1.26 (0.98–1.62)		1.08 (0.84–1.38)	
**Barriers to exercise**												
Lack of facilities	1.02 (0.81–1.30)		1.03 (0.81–1.31)		0.84 (0.60–1.18)		0.78 (0.55–1.09)		1.27 (0.88–1.84)		1.43 (0.99–2.07)	
Lack of money	1.11 (0.89–1.40)		1.04 (0.83–1.31)		1.20 (0.86–1.68)		1.06 (0.75–1.49)		1.02 (0.74–1.41)		1.04 (0.76–1.43)	
Poor health status	1.05 (0.83–1.34)		1.08 (0.85–1.38)		1.31 (0.90–1.89)		1.40 (0.96–2.04)		0.84 (0.60–1.18)		0.83 (0.59–1.16)	
Taking care of children or elderly	0.68 (0.53–0.88)	**	0.72 (0.56–0.92)	*	0.78 (0.52–1.19)		0.77 (0.50–1.17)		0.61 (0.44–0.86)	**	0.64 (0.46–0.90)	*
Lack of time	1.01 (0.79–1.30)		0.81 (0.63–1.05)		0.76 (0.52–0.12)		0.60 (0.41–0.89)	*	1.30 (0.91–1.84)		1.02 (0.72–1.44)	

The reference category of the dependent variable was “no exercise.”

*p <0.05,

**p <0.01,

***p <0.001.

In model 2, we found that male participants living in the suburbs were less likely to exercise irregularly than those living in the city (OR = 0.29; p = 0.041), that those with a higher education level were more likely to exercise regularly or irregularly (ORs were 2.17 and 2.47, respectively; p<0.05), and that the barrier of “having no time for exercise” had a significant relationship with less regular exercise (OR = 0.60; p = 0.011). All of the above factors were not significant in model 3 (female participants).

In model 3, we found that women aged 40–64 years were less likely to exercise regularly (OR = 0.29; p = 0.024) and that those who did not have a spouse or partner were less likely to exercise regularly (OR = 0.47; p = 0.032). Female participants who had better self-rated health were more likely to never exercise than to exercise irregularly (OR = 0.74; p = 0.045), and those feeling it was more worthwhile to spend money on exercise were more likely to have better exercise behavior (ORs were 1.46 and 1.49 for irregular and regular exercise, respectively; p<0.05). As for the barriers to exercise, women who have to take care of others were less likely to exercise regularly or irregularly (ORs were 0.61 and 0.64, p = 0.004 and 0.01 for irregular and regular exercise, respectively).

The only factor that showed influence for both genders was “enjoyment” in doing exercise. A stronger significant influence was found in men (ORs were 3.39 and 6.89 for irregular and regular exercise, respectively; p < 0.001) than in women (OR = 1.82 for irregular exercise, OR = 3.60 for regular exercise; p<0.001).

## 4. Discussion

The main finding of this study was to clarify the gender differences and related influential factors in being physically active; Table 4. summarizes these factors. For male participants, those who lived in the city, had a higher education level, enjoyed exercise more, and did not have the barrier of lack of time were more likely to exercise regularly or irregularly. On the other hand, female participants over 65 years old compared to those aged between 40 to 64 years old, with a spouse or partner, with lower scores of self-rated health, enjoying exercise more, feeling more worthy to spend money on exercise, and not having the barrier of taking care of others were more likely to exercise regularly or irregularly.

**Table 4:** Summarization of the factors that influences exercise behavior.

	Irregular exercise		Regular exercise	
	Facilitators	Barriers	Facilitators	Barriers
**Male**	Higher education level More enjoyment in exercise	Living in the suburbs compared to the city	Higher education level More enjoyment in exercise	Lack of time
**Female**	More enjoyment in exercise Feeling more worthy to spend money on exercise	Better self-rated health Have to take care of others	More enjoyment in exercise Feeling more worthy to spend money on exercise	40–60 years old compared to over 65 years old No spouse or partner Have to take care of others

These factors influence the likelihood of participants to exercise irregularly or regularly rather than “no exercise”.

For male participants, the significance found in living environment and education level was consistent with previous studies [10]. A possible explanation is that they have a better understanding of the benefits of exercise, and can obtain more resources to exercise. “Enjoyment” was found to affect both genders in our current study. Further, we found men who enjoyed exercise more had twice the odds of being regularly engaged in exercise than women. This finding showed that “enjoyment” was a stronger determinant for men than for women. A possible explanation is that 20.2% of the regular exercising male participants in the present study were most often engaged in competitive sports, in comparison, only 5.0% of females reported competitive sports as the activity they most often do. And previous studies have found that men tend to gain more enjoyment in competitive sports in which they can gain mastery, overcome challenges, or gain strength [25]. The only barrier that influenced the male participants was “lack of time”. It is possible that work or activities related to work might occupy most of men’s time, which hinders them from exercising regularly. A study which developed a structural model of physical activity participation for Korean middle-aged adults also pointed out that Korean men exercise for social desirability and fear of disease or aging may affect work [27]. This may be related to the collectivistic culture and emphasis on how other people value oneself in the eastern culture.

In contrast, for female participants, we found those women over 65 years old were more likely to exercise regularly compared to those aged 40–64 years old. This finding concerning age was similar to a cross-sectional Asian study [11]. There is more leisure time to exercise after retirement, however, women in the 40–64 age group needs to spend more time for work or family care. On the other hand, people with more social support tend to be more physically active, and their social support usually come from friends or family. It may be easier for people with a spouse or partner to find company to go exercise together. In terms of health condition, a survey study in Shanghai women pointed out that a history of chronic disease is positively associated with exercise participation [11]. One Malaysian study also found that patients diagnosed with hypercholesterolemia were more likely to be physically active [26]. The findings of our study indicated female participants with lower self-rated health score were more likely to exercise irregularly, suggesting that those who think they were less healthy are more willing to go exercise, but this motivation was not enough for them to engage in exercise regularly or that they are not able to engage in exercise regularly because of poor health.

Few studies have examined how cost will influence exercise behavior. According to the literature, exercise is negatively associated with the costs and travel time required per occasion, including member fees, parking fees, facility charges, and depending on the exercise, the cost of the exercise itself [28]. Rather than the actual money spent on exercise, in this study we examined the perception of how worthwhile it was to spend money on exercise and found that it was only significant for the female participants. A possible explanation is that women are more likely to attend group exercising classes, such as yoga or aerobic dancing, and attending these classes requires class fees. These types of group classes offer various options as a leisure and social activity, which may be an attractive reason to participate, particularly for women.

The only barrier that had a significant effect in the exercise frequency of women was the barrier of “taking care of children or the elderly”. Culture might play an important role in this result. Most Asian families have a deep bond between family members, and it is common to live with parents after adulthood or even after marriage. In a three-generation household, Women typically assume the role of caring for children and the elderly in the family. Our study did not find a significant influence of the barriers “lack of facilities”, “lack of money”, or “poor health status”. This might be because the participants in our study represented a wide range of ages (from 19 to 95) and their health conditions varied widely. The environmental barriers were not as important as the individual barriers. A possible reason for this finding is that over 50% of the participants often walked or jogged for exercise, which require neither a particular facility nor money.

The data used for analysis was retrieved from the 2007 Leisure Time and Sport Questionnaire, thus people’s exercise behavior might have changed over time. Nowadays, women have higher socio-economic status and independency. Therefore, the factor “Feeling more worthy to spend money on exercise” might become a stronger significant facilitator for women. Modern women with higher economic autonomy will have more choices for exercise. The barrier “No spouse or partner”, on the other hand, might become less significant. However, some recent studies still found a significant correlation between received partner support and time of physical activity [29]. Other significant factors found in this study was not influenced by time, such as “Enjoyment in exercise” and factors related with the eastern culture and lifestyle [30, 31]. Gender roles in family and the concept that seniors start to enjoy life after retirement did not change in Asian countries for the last decade [11, 30]. South Asian women were more likely to report family responsibility as a barrier to physical activity because they are often regarded as primary care givers [30]. Moreover, there were no consistent conclusion about how other factors that changed in the past decade affected physical activity participation, such as the use of smart phones [32–34]. Based on the above reasons, we believe that the findings of this study still have reference value in modern Taiwanese society. Future studies are needed to examine if the influential factors have changed over time and other factors such as the time of using technological devices can be included in the analysis.

The factors that significantly affected exercise frequency of men and women were different. This can help the development of gender-specific health promotion strategies. Men’s exercise behavior was greatly affected by the enjoyment they gain in exercise and were likely also influenced by the type of exercise performed, but they were significantly hindered by having no time. Therefore, the government can encourage corporations to create an accessible exercising environment, such as a gym or ball court, for their employees and allow them to take at least 30 minutes of work-time off three days a week to exercise. Moreover, companies can arrange contests between departments or colleagues to create competition or challenge. Regarding health promotion strategies for women, health promoting agencies can design group courses, such as yoga or aerobic dancing, and provide temporary day care services during the course to help women who are caregivers. Members can provide support and encouragement to each other to increase confidence. Additionally, to promote such courses, a celebrity with a healthy public image could be invited to be a spokesperson to provide further stimulation and motivation to attend.

There were some limitations to this study. First, the time lapse from the data collected to the present day. The Sports Department of the Ministry of Education, Taiwan stated that the proportion of women engaging in regular exercise has caught up with men in 2018. Satisfaction with the government’s public sports facilities has also increased in recent years. Second, the participants were only in Taiwan, Asia. Therefore, it may be difficult to generalize the findings to western countries. Cultural difference may influence the determinants of being physically active. Third, the factors included in the analysis were drawn from secondary data. Thus some variables were unavailable in the dataset, such as knowledge of health and exercise self-efficacy. Forth, it was rather difficult to divide the exercise types into five clear categories according to the participant’s own description of the type of exercise most commonly performed. The “Walking” group may diverse in its intensity according to the paces of the participants. Moreover, the actual time of the exercise was not recorded. We assume the time for competitive sports and aerobic exercising was at least more than 30 minutes, which means the intensity was able to increase heart rate and improve cardiopulmonary capacity.

## 5. Conclusions

There were gender differences in regular exercise participation and related influential factors among people in Taiwan. These findings give health promoters a clear picture of the type of men and women more likely to be physically active and more willing to perform exercise regularly. This can be used for future interventions and policies. Future research is needed to find the causal relationship between these factors and the frequency of exercise to further support our results. Among the factors, we are particularly interested in the different barriers that hinder men and women. Longitudinal studies can be conducted to analyze whether there is an increase in the frequency of exercise participation in companies that give employees time off to exercise, and whether the impact on men is more significant than that of women. In addition, in Taiwan, there are already some companies and department stores providing temporary care services. Future studies can be made to investigate whether the sports centers with this service can increase the participation of their members, and whether the impact on women is more significant. In addition, more factors relevant to exercise behavior, for example health knowledge and self-efficacy, should be included in the models, and age groups can be analyzed separately.

## Supporting information

S1 Questionaire (PDF)

S2 Questionaire (PDF)

S1 Data (XLSX)
