# Supplementary material for: Gender differences in related influential factors of regular exercise behavior among people in Taiwan in 2007: A cross-sectional study
Source: PLoS One. 2020 Jan 31;15(1):e0228191. doi: 10.1371/journal.pone.0228191 (PMC6993962; doi:10.1371/journal.pone.0228191)
Supplement: S1 Questionaire — (PDF) [file pone.0228191.s001.pdf]

Leisure Time and Sport Questionnaire

Respondent number：________________________

Time of interview:____________________（24-hour system）

A. Basic data

A1.gender：☐(01)male☐(02)female

A2. Birthday: ________________________

A3. Where do you currently live?

_________Province_________County（City） _________Township（Town, District）

Postal code☐☐☐

A4. The area you live in is urban or rural?

☐(01)Big City☐(02)The suburbs of a big city☐(03)Little town☐(04)Rural area☐(05)Independent farm

A5. Where is your father from?

☐(01) Taiwanese Minnanese ☐(02) Taiwanese Hakkas ☐(03) Mainland China☐(04) Taiwan Aboriginal☐(05) Southeast Asia ☐ (06) Others, please explain _____

A6. What is your father's education level?

☐(01)Illiterate ☐(02)Self-educated ☐(03)Primary school☐(04)junior high school☐(05)Vocational junior high school☐(06)High School General Section☐(07)High School Vocational Education ☐ (08)Vocational high school☐(09)Sergeant School☐ (10) Five-year vocational school ☐(11) Two-year vocational school after high school (12) Three-year vocational school after high school☐(13) Military and police special classes ☐(14) Military and police school☐(15) (16) Air University ☐ (17) Military Police University☐(18) Technical College ☐(19) University ☐(20) Master☐(21)Phd ☐(22) Others, please specify:____________

A7 Does your father have a job since you were born?

☐(01)Yes, always had a full-time job.☐(02)Yes, always had a part-time job.

☐(03)Sometimes yes, sometimes no.☐(04)Never had a job.

A8. Where is your mother from?

☐(01) Taiwanese Minnanese ☐(02) Taiwanese Hakkas ☐(03) Mainland China☐(04) Taiwan Aboriginal☐(05) Southeast Asia ☐ (06) Others, please explain _____

A9. What is your mother's education level?

☐(01)Illiterate ☐(02)Self-educated ☐(03)Primary school☐(04)junior high school☐(05)Vocational junior high school☐(06)High School General Section☐(07)High School Vocational Education ☐ (08)Vocational high school☐(09)Sergeant School☐ (10) Five-year vocational school ☐(11) Two-year vocational school after high school (12) Three-year vocational school after high school☐(13) Military and police special classes ☐(14) Military and police school☐(15) (16) Air University ☐

(17) Military Police University☐(18) Technical College ☐(19) University ☐(20) Master☐(21)Phd ☐(22) Others, please specify:____________

A10. Does your mother have a job since you were born?

☐(01)Yes, always had a full-time job.☐(02)Yes, always had a part-time job.

☐(03)Sometimes yes, sometimes no.☐(04)Never had a job.

A11. What is your education level?

☐(01)Illiterate (skip to question A14)☐(02)Self-educated (skip to question A14)☐(03)Primary school☐(04)junior high school☐(05)Vocational junior high school☐(06)High School General Section☐(07)High School Vocational Education ☐(08)Vocational high school☐(09)Sergeant School ☐ (10) Five-year vocational school ☐(11) Two-year vocational school after high school (12) Three-year vocational school after high school☐(13) Military and police special classes ☐(14) Military and police school☐(15) (16) Air University ☐(17) Military Police University☐(18) Technical College ☐(19) University ☐(20) Master☐(21)Phd ☐(22) Others, please specify:____________

A12.Did you graduate？

☐(01)No, incomplete☐(02)No, still in school☐(03)Yes

A13.How many years of education did you have in total?

A14.How is your current marital status?

☐(01)Married and have a spouse（skip to question A16） ☐(02)Widow or widower☐ (03)divorced ☐(04)separated but not divorced ☐(05)single, never married before☐(06)other situation, please explain:___________

A15 Do you currently have a partner living together with you? ☐(01)Yes☐(02)No

A16.Do you currently have a religion?

☐(01) Buddhism ☐ (02) Taoism ☐ (03) Folk beliefs☐ (04) Yikuanism ☐ (05) Islam ☐ (06) Catholic☐ (07) Christian ☐ (08) No religious beliefs ☐ (09) Others, please explain: ______

A17.How often do you attend religious activities? （Don't answer this question if you have no religious beliefs.）

☐(01)Several times a week☐(02)Once a week☐(03)2-3 time per month☐(04)Once a month☐ (05)Several times a year☐(06)Once a year☐(07)Barely attend those activities（Less than once a year） ☐(08)Never attend

A18. Do you currently have a job?

☐(01)Yes, full-time☐(02)Yes, part-time☐(03)No fixed job, currently don't have a job☐(04)helping with the family business☐(05)No job（skip to A20）☐(06)currently a student with no job（skip to A20）☐(07)retired☐(08)housewife with no job（skip to A20）☐(09) elderly, have physical and mental disabilities or illness, unable to work (skip to A20) ☐ (10) Others, please explain:_____________

A19.What work do you do?（If you are retired, please fill in the information of your previous work.）

a. Company name: ____

Main product or service:____ Industry:□□□

b. Department: ___________________, Position: ____________________

Detailed work content:____ISCO88□□□

c. How many hours per week do you work in average? _____________hours

d. Do you manage other employees in your work? □(01)Yes □(02)No

e. What is the nature of your work?

□(01)Working in government departments (skip to A20)

□(02)Working in public enterprises (skip to h)

□(03) Opening your own business

□(04) work of other nature (skip to h)

f. Do you have any employees? □(01)Yes□(02)No (skip to h)

g. How many employees do you hire? ____________

h. Have you joined the work union now or before?

□(01)Yes, now. □(02) Yes, before. But not now. □(03) never joined

＜Currently single and without a partner living together, please skip to A22; Widow or widower skip to A21＞

A20. Does your spouse (cohabiting partner) work now?

□(01)Yes, full-time□(02)Yes, part-time□(03)No fixed job, currently don't have a job□(04)helping with the family business□(05)No job（skip to A22）□(06)currently a student with no job（skip to A22）□(07)retired□(08)housewife with no job（skip to A22）□(09) elderly, have physical and mental disabilities or illness, unable to work (skip to A22) □ (10) Others, please explain:______________

A21. What work do your spouse (cohabiting partner) do? （If he/she is retired, please fill in the information of his/her previous work.）

c. Company name: ____

Main product or service:____ Industry:□□□

d. Department: ___________________, Position: ____________________

Detailed work content:____ISCO88□□□

c. What is the nature of his/her work?

□(01)Working in government departments □(02)Working in public enterprises

□(03) Opening your own business□(04) work of other nature

A22. How many people live in your home, including you? (a) ______________ people,

How many of them are over 65 years old (including you)? (b) _______, how many are under 6 years old? (c) _______

B. Attitude of life

How much do you think the statements below matches with your thoughts?

	Totally matches	Somewhat matches	In between	Doesn't really match	Does not match at all	Can't choose
B1. I feel very boring when I always see familiar faces.	01	02	03	04	05	97
B2. Sometimes I like to do something a little risky.	01	02	03	04	05	97
B3. I want to have some adventure alone.	01	02	03	04	05	97
B4. I want to have adventures a lot of other people.	01	02	03	04	05	97

Do you think the following are considered leisure time activities?

	Absolutely yes	Perhaps yes	Perhaps no	Absolutely no	Can't choose
B5. Do things that make you happy.	01	02	03	04	97
B6. Do things that make you relax.	01	02	03	04	97
B7. Do nothing at all.	01	02	03	04	97
B8. Do something for others without asking any return.	01	02	03	04	97
B9. Do things that you want.	01	02	03	04	97
B10. Killing time.	01	02	03	04	97
B11. Do things that have nothing to do with work.	01	02	03	04	97

Leisure time: activity and satisfaction

The following questions are about your personal "free time", which refers to the time you can do things according to your own will, apart from work, housework, and other necessary daily activities.

C1 In your free time, how often do you engage in the following activities?

	Everyday	Several times a week	Several times a month	Several times a year or less	Never
a. Watch TV, DVD, or videos.	01	02	03	04	97
b. Go to the cinema for movies.	01	02	03	04	97
c. Go shopping for recreation.	01	02	03	04	97
d. Read books.	01	02	03	04	97
e. Attend art and cultural activities, such as concerts, theater performances, exhibitions.	01	02	03	04	97
f. Family gathering with relatives that doesn't live with you.	01	02	03	04	97
g. Gathering with friends.	01	02	03	04	97
h. Play cards or chess.	01	02	03	04	97
i. Listen to music.	01	02	03	04	97
j. Engage in physical activities, such as sports, going to the gym, walking, etc.	01	02	03	04	97
k. Go watch sport competitions.	01	02	03	04	97
l. Doing craftwork, such as tailoring, carpentry.	01	02	03	04	97
m. Use computer or go online.	01	02	03	04	97

C2 Can the activities you do in your free time help you?

	Very helpful	Quite helpful	A little helpful	Not very helpful	No help at all	Can't choose
a. Show your true self.	01	02	03	04	05	97
b. Improve interpersonal relationships.	01	02	03	04	05	97

C2a1. Do you usually exercise by yourself or with company (family or friends)?

☐ (01) always with company ☐(02) mostly with company☐ (03) 50% with company

☐(04) mostly alone☐ (05) Always alone (skip to C2a3)

C2a2 When you are doing leisure time activities, who are you usually with?

☐(01) Spouse ☐ (02) Parents ☐ (03) Children ☐ (04) Other family members or relatives

☐(05) Colleagues ☐(06)Neighbors ☐(07)Friends ☐(08)Others, please explain _______

C2a3 In the last three years, how many people will you encounter each time (at most) when you do

your leisure time activities?

□(01) 0-4 people□(02) 5-19 people□(03) 20-99 people□(04) over 100 people

C2a4 In the last three years, how many friends did you make through doing your leisure time activities?

□(01)0 □(02)1-4 people□(03) 5-19 people□(04) over 20 people

C2a5. Do you want to make friends from doing these leisure time activities?

□(01) yes, very much.□(02)a little□(03)don't want so much□(04)don't want at all (don't care)

C3 How much enjoyment do you gain from doing these activities in your leisure time?

	Do not enjoy at all	Not so much	Some	Fairly enjoy	A lot of enjoyment	Never do these activities
a. Reading books	01	02	03	04	05	97
b. Gathering with friends	01	02	03	04	05	97
c. Engage in physical activities, such as sports, going to the gym, walking, etc.	01	02	03	04	05	97
d. Watch TV	01	02	03	04	05	97

The meaning of time and leisure activities, and their relationship with work and other areas of life

C4 Do you often use free time to do the following activities?

	Always	Often	Sometimes	Rarely	Never	Can't choose
a. Build useful networks with people.	01	02	03	04	05	97
b. Relax and regain energy.	01	02	03	04	05	97
c. Try to learn or improve skills	01	02	03	04	05	97

C5A.In your leisure time, do you have the feelings below?

	Always	Often	Sometimes	Rarely	Never	Can't choose
a. Boring	01	02	03	04	05	97
b. In a hurry	01	02	03	04	05	97
c. Find oneself thinking about work	01	02	03	04	05	97

C5B. In your free time, do you prefer to be with others, or do you like to be with yourself?

□(01) always likes to be with others.

□(02) prefer to be with others, better than being alone.

□(03) prefer to be alone, better than with others.

□(04) always like to be alone □(05) can't choose

C6. Suppose you can reschedule your time. Do you want to spend more time or less time on these

things compared to now?

	Much more	More	Same	Less	Much less	Can’t choose
a. Working to earn money	01	02	03	04	05	97
b. Do housework	01	02	03	04	05	97
c. Spend time with family	01	02	03	04	05	97
d. Leisure time activities	01	02	03	04	05	97

C7a. In the past year, how many nights have you spent out on vacation or gathering with friends?
□(01)always at home□(02)1-5 nights□(03)6-10 nights□(04)11-20 nights□(05)21-30 nights□(06)over 30 nights□(07)Can’t choose

C7b. In the past year, how many days did you take off without working? (excluding public holidays, holidays, maternity leave, parental leave, sick leave, ect.)

Sports/games and their subjective functions

C8a.What sports/exercise/physical activity do you most often do? _________________ N0.□□

C8a1. If you don’t do this exercise or physical activity, for how long will you start to feel uncomfortable or not quite right?
□(1) 1 day□(2)2-3 days□(3)1 week□(4)2 weeks□(5)1 month or more□(6)Never had this feeeling

C8a2. What is your second often done sports/exercise/physical activity? ______________NO.□□
□(01)I don’t have a second sports/exercise/physical activity

C8b. What games do you most often play in addition to sports or physical activity?

<u>Board/Card games</u>

□01 Western double chess game
□02 checkers
□03 chess
□04 Go
□05 Other board games (eg. monopoly, English scrabble)
□06 card games (eg. bridge, ghost sparrow, etc.)
□07 Domino
□08 Mahjong
□09 Puzzle
□10 text or slang games (eg. crossword puzzle)
□11 Video games, computer games, PS TV cruiser, marble ball game
□12 gambling games (eg casino games, slot machines, sports gambling)
□13 Special games in various countries (chess)
□14 Others：

□15 I don’t play any games

C9. Is the following reason for participating sports or games important to you?

	Very important	A little	Not so important	Not important	Can’t choose	Not applicable
a. For health	01	02	03	04	97	99
b. Get connected with other people	01	02	03	04	97	99
c. Competing with others	01	02	03	04	97	99
d. For a better figure	01	02	03	04	97	99
e. For interest	01	02	03	04	97	99

C10a What kind of sports competitions do you most often go to or watch on TV?

____________________ NO.□□□

□(01) I don’t watch any sports competitions（skip to C11）

C10b What second kind of sports competitions do you often go to or watch on TV?

________________ NO.□□□

□(01) I don’t have a second often watched sports competitions

Sociological perspective of sports and leisure activities

C11. When Taiwanese athletes perform well in international sports competitions, do you feel honored?

□(01)Yes, very honored□(02)A little□(03)I don’t feel very honored□(04)Not honored at all

□(05)Can’t choose

C12. Do you agree with the following statements?

	Strongly agree	Agree	In be-tween	Don’t agree	Strongly disagree	Can’t choose
a. Participating in sports will help children’s personality development.	01	02	03	04	05	97
b. There are too many sports shows on TV.	01	02	03	04	05	97
c. Sports can bring different groups and races in the country closer.	01	02	03	04	05	97
d. International sports competitions are more likely to cause tensions between countries	01	02	03	04	05	97
e. The government should allocate more funds to sports.	01	02	03	04	05	97

Social and political participation

C13 In the past year, how often do you participate in the activities of these groups?

	At least once a week	At least once a month	Several times	1-2 times	Never
1. Sports Association / Group	01	02	03	04	05
2. Cultural associations/groups	01	02	03	04	05
3. Church or other religious organization	01	02	03	04	05
4. Community services or general civil society/groups	01	02	03	04	05
5. Political parties or political organizations	01	02	03	04	05

C14a.Do you think that you can trust most people or you should be careful about other people?

□(01) You can always trust □ (02) You can usually trust most people□ (03) usually must be careful □ (04) always have to be careful□(05) cannot choose

C14b How interested are you in politics?

□(01) Very interested□(02) a little interested□(03) not very interested□(04) Not interested at all. □(05) cannot choose

Social determinants of leisure activities

C15. How much would the following factors influence the frequency of your exercise during leisure time?

	Very strong influence	Strong influence	A little	No influence at all	Can't choose
a. Lack of facilities	01	02	03	04	05
b. Lack of money	01	02	03	04	05
c. Poor health status	01	02	03	04	05
d. Taking care of children or the elderly	01	02	03	04	05
e. Lack of time for exercise	01	02	03	04	05

C16 Overall, how happy would you say you are?

□(01)Very happy□(02)happy□(03)Not so happy□(04)Not happy at all□(05)Cannot choose

C17. In general, how do you think of your health condition?

□(01)Excellent□(02)Very good□(03)Good□(04)Fair□(05)Poor□(06)Cannot choose

D Sports

D1 Do you agree that your child will become a professional athlete? (If you have a child)

☐(01) Very much agree (02) Agree☐(03)Don’t agree very much ☐(04) Disagree

D2. Which is more suitable for describing the "boxing" sport?

☐(01) Powerful but needs a lot of skills☐ (02) Rough and brutal☐(03) No opinion (04) Others, please specify: ______

D3 Which is more suitable for describing the "golf" sport?

☐(01) noble☐ (02) expensive☐(03) No opinion (04) Others, please specify: ______

D4 What do you think of the physical collision in a basketball game?

☐(01) Reasonable competitive strategy ☐ (02) normal and cannot be avoided☐(03) Rude behavior that should be avoided ☐ (04) No opinion☐(05)Other, please specify: _______

D5 If an opponent accidentally hits a person during a sports match, do you think you can hit it back?

☐(01) must hit back ☐(02) probably☐(03) probably not ☐(04) must not☐(97) don’t know

D6 Do you think that it is a reasonable psychological strategy to swear to your opponent in the sports competition?

☐(01) must do that☐ (02)probably yes☐(03) probably not ☐(04) must not do that☐(97) don’t know

D7 Do you feel that it is worthwhile to spend money on exercise?

☐(01)Very worthwhile☐(02)Fairly worthwhile☐(03)Not so worthwhile☐(04)Not worthwhile at all

D8 Do you agree that exercising means just moving is enough, you don’t have to push yourself too much?

☐(01) Strongly agree ☐(02) Agree☐(03) Don’t agree or disagree ☐(04) Disagree☐(05) Strongly disagree

D9 Do you think the following activities are considered sports?

	Strongly agree	Agree	In between	Don’t agree	No opinion	Don’t know
a. Tai-Chi	01	02	03	04	05	97
b. Qi-gong	01	02	03	04	05	97
c. Song Jiang array	01	02	03	04	05	97
d. Yoga	01	02	03	04	05	97

D10 When you determine whether an activity is or is not sports, are following conditions important?

	Very important	A little	Not so important	Not important	No opinion	Don’t know
a. The competitive nature of the activity	01	02	03	04	05	97
b. Clear rules	01	02	03	04	05	97
c. The amount of physical activity is large enough	01	02	03	04	05	97
d. Maintain health	01	02	03	04	05	97

e. Body building	01	02	03	04	05	97

E Tourism

E1 Do you like the following types of sightseeing trips?

	Like very much	Like	A little	Don't like	Don't know	Refuse to answer
a. Outdoor adventures (such as boating, skiing, rock climbing)	01	02	03	04	05	97
b Historical artifacts (eg museums, art museums)	01	02	03	04	05	97
c Fixed-point holiday (eg resort, leisure hotel)	01	02	03	04	05	97
d View natural scenery (eg Grand Canyon, Waterfall)	01	02	03	04	05	97
e. Shopping (such as department stores, boutiques)	01	02	03	04	05	97

E2 How do you usually arrange your domestic travel itinerary?

☐(01) Never attend (skip to E4)

☐(02)Attend friends', companies', associations', or communities' tour group

☐(03)Sign up to the travel agency and join the tour group

☐(04) free travel, self-service travel

☐(05) Others, please specify: ____________________

E3 In the past three years, how many times have you been sightseeing in Taiwan? ___ times

E4 Do you want to travel in Taiwan recently?

☐(01) I really want to ☐(02) A little☐(03)Not very much (04) I don't want to

☐(97) don't know ☐(98) refused to answer

E5 How do you arrange your travel abroad?

☐(01) Never attend (skip to E7)

☐(02)Attend friends', companies', associations', or communities' tour group

☐(03)Sign up to the travel agency and join the tour group

☐(04) free travel, self-service travel

☐(05) Others, please specify: ____________________

E6 How many times have you traveled abroad in the past three years? ___ times

E7 Do you wanted to travel abroad recently?

☐(01) I really want to ☐(02) A little☐(03)Not very much (04) I don't want to

☐(97) don't know ☐(98) refused to answer

E8 Work and leisure, which one do you think is more important?

☐ (01) work ☐ (02) leisure

E9 How would you arrange your work time and the income you make?

☐(01) Work longer hours in order to make more money

☐(02) work the same time, earn the same money

☐(03) It’s okay to work shorter and earn less.

☐(97)I don’t know, can’t choose

E10 Can you decide how to arrange your daily work?

☐(01) can be freely decided ☐ (02) I can decide more or less

☐(03) can't decide freely ☐(04) can't choose

F Leisure time activity dispute resolution and security

F1 Have you or your family had any disputes or unhappiness with the travelling industry in the past 10 years?

☐(01) There were major disputes ☐(02) There were minor disputes☐(03) There was a little unpleasantness ☐(04)No

F2 If there is a major loss in your money caused by a dispute when you do your leisure time activities, but the company is not willing to pay compensation, which one of the following personal or organization do you think will best help you to seek compensation?

☐(01) Court

☐(02) Prosecutor

☐(03) Lawyer

☐(04) Township (town, city, district) mediation committee

☐(05) Government Consumer Protection Committee (COP)

☐(06) Folk Consumer Culture and Education Foundation (COF)

☐(07) Insurance company insured by the industry

☐(08) You (the consumer) insured insurance company

☐(09) public representative

☐(10)Media

☐(11) Others, please specify:_______

F3 Continuing the previous question. If it caused serious harm to your body, which of the following personal or organization do you think will best help you with your claim?

☐(01) Court

☐(02) Prosecutor

☐(03) Lawyer

☐(04) Township (town, city, district) mediation committee

☐(05) Government Consumer Protection Committee (COP)

☐(06) Folk Consumer Culture and Education Foundation (COF)

☐(07) Insurance company insured by the industry

☐(08) You (the consumer) insured insurance company

□(09) public representatives
□(10)Media
□(11) Others, please specify: ______
F4 If accidents such as natural disasters and earthquakes occur in private leisure activities, causing major losses on consumer's money, but the company are not at fault, who do you think should be mainly responsible for the situation?
□(01) Consumers themselves □ (02) The company providing service
□(03) Government□ (04) The company providing service and the government
□(05) Insurance company insured by the industry
□(06) You (the consumer) insured insurance company
F5 Continuing the previous question, if someone is injured or killed, who do you think should be mainly responsible for the situation?
□(01) Consumers themselves □ (02) The company providing service
□(03) Government□ (04) The company providing service and the government
□(05) Insurance company insured by the industry
□(06) You (the consumer) insured insurance company
F6 Do you think that the country should make laws for leisure and entertainment venues to provide special services for those with limited mobility. Otherwise, they should be punished?
□(01) Absolutely necessary □(02) Should be necessary□(03) Not so necessary □(04) Absolutely not necessary
F7 In general, the monthly or annual ticket fee for a swimming pool is the same for men and women. If you give women a cheaper fare or a longer period of use because of women's physiological factors, do you think this is unreasonable?
□(01) Very reasonable □(02) Reasonable□(03) Not reasonable □(04) Very unreasonable
F8 The locker at the entertainment venues is marked "Do not place valuables, if lost, there will not be compensation." Do you think this is unreasonable for consumers?
□(01) Very reasonable □(02) Reasonable□(03) Not reasonable □(04) Very unreasonable
F9 When you go to the cinema or KTV, will you check or pay attention to the safety of its fire protection facilities?
□(01) will definitely □(02) sometimes□(03) rarely will □(04) certainly not
□(99) not applicable/had never been
F10 Do you think the government will regularly check the fire protection facilities of "cinema or KTV"?
□(01) will definitely □(02) will probably□(03) probably won't □(04) will not
F11 Do you think the government has actually checked the fire protection facilities of "cinema or KTV"?
□(01) Must have □(02) Sometimes □(03) Rarely □(04) Must have not
F12 Do you think that the amusement park "will regularly check" the safety of their facilities?

□(01) will definitely □(02) will probably□(03) probably won't □(04) will not

F13 Do you think that the amusement park “has indeed checked and maintained” the safety of their facilities?

□(01) They must have □(02) Sometimes□(03) Rarely □(04) Must not

F14 When you go to a leisure farm or a homestay, will you check or notice if it has a government-certified certificate?

□(01) will definitely □(02) sometimes□(03) rarely will □(04) certainly not

□(99) not applicable/had never been

F15 When you join a tour group of a travel agency, will you read the contract content?

□(01) Yes, read it carefully □ (02) Yes, will browse over it □(03) No, will only ask the travel agency□(04) No, listen directly to the staff of the agency□(05) No, I let my relatives or friends to decide for me.

F16 Do you think that the contract signed with the travel agency can effectively protect your rights when dispute occurs?

□(01) must be □(02) probably□(03) probably not □(04) must not□(98) Refuse to answer

F17 Generally, travel agencies have already insured the comprehensive tourism liability insurance in accordance with the law before the trip. Do you know who’s benefit is this for?

□(01) I don’t know this insurance.

□(02) I know. That is for the member.

□(03) I know. That is for the travel agency.

□(04) I know. That is both for the member and the travel agency.

□(05) I don’t know it’s for who’s benefit.

G Leisure time and reading

G1 In the past three years, how much time do you spend on reading per week? (excluding reading newspapers and magazines) ______hours______minutes

□(1)Never read books（skip to H1）

G2 In your free time, do you often read the following types of books?

	Often	Sometimes	Seldom	Never
a. Romance novels	01	02	03	04
b. Martial arts, detectives, fantasy, etc. novels	01	02	03	04
c. Family life (DIY, health, education)	01	02	03	04
d. Entertainment / fashion	01	02	03	04
e. Art Culture / Travel	01	02	03	04
f. Finance / Economics	01	02	03	04
g. Science / Information Technology	01	02	03	04
h. Spiritual inspiration /Religion	01	02	03	04

i. Classical Literature / Modern Literature	01	02	03	04
j. Biography / History	01	02	03	04
k. Bestsellers	01	02	03	04
l. Comics	01	02	03	04
m. Others, please specify: ____________	01	02	03	04

G3 According to your experience in reading books, do you agree with the following statements?

	Strongly agree	Somewhat agree	Somewhat disagree	Strongly disagree
a. Reading brings me joy in life.	01	02	03	04
b. Reading increases my knowledge.	01	02	03	04
c. Reading adds more topics to my conversation when I interact with others.	01	02	03	04
d. Reading helps me relax.	01	02	03	04

H. Personality and life experience

H1Do you worry about how to arrange your time on holidays?

□(01)Often□(02)Sometimes□(03)Seldom□(04)Almost never

H2 In the past month, does your leisure time activities bring you the following feelings?

	Often	Sometimes	Seldom	Never
a. My leisure activities are very interesting to me.	01	02	03	04
b. My leisure activities have increased my knowledge of the things around me.	01	02	03	04
c. I can interact with others through leisure activities.	01	02	03	04
d. My leisure activities help me relax	01	02	03	04
e. My leisure activities help me stay healthy	01	02	03	04
f. My place for leisure activities is well designed.	01	02	03	04

H3 Do you think the following characters match your personality?

	Strongly agree	Somewhat agree	Somewhat disagree	Strongly disagree
a. don’t like to talk	01	02	03	04
b. very compassionate	01	02	03	04
c. never leave work to the next day	01	02	03	04
d. know how to relax and deal with stress	01	02	03	04
e. imagination is rich	01	02	03	04

f. outgoing, good at networking with people	01	02	03	04
g. don't trust others	01	02	03	04
h. careless	01	02	03	04
i. get nervous easily	01	02	03	04
j. is a conservative person	01	02	03	04

H4 Do you have any of the following experiences or feelings?

	Often	Sometimes	Seldom	Never
a. want to take advantage of others.	01	02	03	04
b. jealous about other people being lucky.	01	02	03	04
c. talk about other people behind their backs.	01	02	03	04

H5 How many people do you usually make contact with in a normal day? (refers to one-on-one contact, including nodding, greeting, speaking, making a phone call, writing a letter, etc.)

□(01)0-4 people □(02) 5-9 people □(03)10-19 people

□(04)20-49 people □(05)50-99 people □(06)100 or more

H6 When you listen to music, what type of music do you like the most? (Select at most three items)

□(01)Do not listen to music

□(02) Taiwanese Opera

□(03) Traditional Chinese Opera (or other local opera)

□(04) Traditional Chinese music

□(05)Taiwanese old songs

□(06)Taiwanese pop songs

□(07) Mandarin Old Songs

□(08) Mandarin Pop Songs

□(09) Western Old Songs

□(10) Western Pop Songs

□(11)Japanese pop songs

□(12)Korean pop songs

□(13) Classic music (including opera)

□(14) Blues or jazz

□(15) rock or hip hop

□(16) musicals

□(17)Others, please specify ______

H7 What kind of food do you like the most? (Select at most three items)

□(01) Taiwanese cuisine

□ (02) dishes from all provinces of China(such as Sichuan cuisine, Jiangsu and Zhejiang cuisine)

□(03) Hakka cuisine

☐(04) American cuisine (such as steak, burger)

☐(05) European cuisine(eg French, German, Italian)

☐(06)Japanese cuisine

☐(07)Southeast Asian cuisine(such as Thai food, Vietnamese food)

☐(08) Others, please specify ______

H8 Where do you like to go when you go out to eat?

☐(01) Roadside booth☐(02) snack bar

☐(03) department store or Food court in the mall☐(04)Special restaurant Or fine dining restaurant

☐(05)Others, please specify _____

H9 How many days in a week do you usually have dinner outside? days

H10 When do you usually get up?

H11 When do you usually go to sleep?

H12 When the police caught the suspect in a criminal case, do you think it's reasonable for the victim (or his family) to beat up the suspect?

☐(01) is very reasonable ☐(02) somewhat reasonable☐(03) is not very reasonable ☐(04) very irrational☐(05) no opinions about this

H13 Do you think that hiring a gangster to help with debt collection will be more effective than a lawsuit?

☐(01) will definitely ☐(02) probably☐(03) probably won't ☐(04) will not☐(97) don't know

I. Income

I1. At present, there are several major political parties in the country, including the Kuomintang, the Democratic Progressive Party, the People First Party, the New Party, etc. Are you more inclined to one of the political parties?

☐ (01) Kuomintang ☐ (02) DPP ☐ (03) New Party☐ (04) People First Party ☐ (05) Taiwan Party (06) without party membership☐ (08) Not clinging to any party ☐ (07) Others, please explain ______

I2. If the society is divided into ten stratums from top to bottom, the first level represents the lowest social status and the tenth level represents the highest level. Which level do you think matches your social status?

I3. What is your average monthly income before tax (pre-tax)? (including salary, year-end bonus, annual bonus, overtime pay, executive business income, self-employed income, investment interest, rent, pension, or parent/child Living expenses and other income)

☐(01)No income ☐(02)Under 10,000 NTD

☐(03) 10,000-20,000 NTD or less ☐(04) 20,000-30,000 NTD or less

☐(05)30,000-40,000 NTD or less ☐(06)40,000-50,000 NTD or less

☐(07)50,000-60,000 NTD or less ☐(08)60,000-70,000 NTD or less

☐(09)70,000-80,000 NTD or less ☐ (10) 80,000-90,000 NTD or less

☐(11)90,000-100,000 NTD or less ☐(12)100,000-110,000 NTD or less
☐(13)110,000-120,000 NTD or less ☐(14)120,000-130,000 NTD or less
☐(15)130,000-140,000 NTD or less ☐(16)140,000-150,000 NTD or less
☐(17)150,000-160,000 NTD or less ☐(18)160,000-170,000 NTD or less
☐(19)170,000-180,000 NTD or less ☐(20)180,000-190,000 NTD or less
☐(21)190,000-200,000 NTD or less ☐(22)200,000-300,000 NTD or less
☐(23)more than 300,000 NTD

I3. What is your average whole family income before tax (pre-tax)? (including salary, year-end bonus, annual bonus, overtime pay, executive business income, self-employed income, investment interest, rent, pension, or parent/child Living expenses and other income)

☐(01)No income ☐(02)Under 10,000 NTD
☐(03) 10,000-20,000 NTD or less ☐(04) 20,000-30,000 NTD or less
☐(05)30,000-40,000 NTD or less ☐(06)40,000-50,000 NTD or less
☐(07)50,000-60,000 NTD or less ☐(08)60,000-70,000 NTD or less
☐(09)70,000-80,000 NTD or less ☐(10) 80,000-90,000 NTD or less
☐(11)90,000-100,000 NTD or less ☐(12)100,000-110,000 NTD or less
☐(13)110,000-120,000 NTD or less ☐(14)120,000-130,000 NTD or less
☐(15)130,000-140,000 NTD or less ☐(16)140,000-150,000 NTD or less
☐(17)150,000-160,000 NTD or less ☐(18)160,000-170,000 NTD or less
☐(19)170,000-180,000 NTD or less ☐(20)180,000-190,000 NTD or less
☐(21)190,000-200,000 NTD or less ☐(22)200,000-300,000 NTD or less
☐(23)300,000-400,000 NTD or less ☐(21)400,000-500,000 NTD or less
☐(22)500,000-1000,000 NTD or less☐(23)more than 1000,000 NTD

End of interview: _____ month _____ day _____ hour _____ minutes

Respondents' phone number: ( ) _____________________; mobile phone ____________________

J Interview record

J1. Interviewer ID: ___________

J2. Did you complete the interview in one visit?

☐(1)Yes

☐(2)No (please fill in the following visit start and end times)

First start time: _____ month _____ day _____ hour _____ minutes; end time: _____ hour _____ minutes

The second start time: _____ month _____ day _____ hour _____ minutes; end time: _____ hour _____ minutes

The third start time: _____ month _____ day _____ hour _____ minutes; end time: _____ hour _____ minutes

J3. At the time of the official visit, this questionnaire is:

☐(01)Respondents fill in by self-selves

☐(02)Interviewer fill in the questionnaire, but did not give the respondent a questionnaire to look at

☐(03) Interviewer fill in the questionnaire, but also gave the respondent a questionnaire to look at

☐(04) Interviewed the respondent with the help of a third party interpreting

☐(05)Others, please specify _______________

J4. During the interview, did the respondent say that they refused to be interviewed?

☐(01) refused at the beginning

☐(02) rejected during the visit.

☐(03) rejected at the end of the visit

☐(04) have rejected many times from beginning to end of the interview

☐(05) did not indicate rejection from beginning to end

J5. During the interview, did the respondent express impatience?

☐(01) never ☐(02) occasionally expressed impatience

☐(03) Sometimes impatient ☐(04) Always impatient

J6. How much did the respondent trust the interviewer during the visit?

☐(01) very low ☐(02) low☐(03)High ☐(04) very high

J7. During the interview, did the respondent treat the interviewer casually?

☐(01) Most of the time ☐ (02) Sometimes ☐(03) rarely ☐(04)never

J8. Cooperation of the respondent:

☐(01) very cooperative ☐(02)cooperative☐(03) does not cooperate ☐(04) very uncooperative

J9. The reliability of the respondent:

☐(01) Very reliable ☐(02)Reliable☐(03) Unreliable ☐(04) very unreliable

J10. The language used for access is:

☐(01)Mandarin ☐(02)Taiwanese☐ (03) Hakka ☐ (04) Guotai☐ (05) National Hakka ☐ (06) Others, please explain____________

J11. Did the interviewer work independently when interviewing the respondent? ☐(1)Yes ☐(2)No

J12. Is there a prior appointment made before the visit? ☐(1) is ☐(2)No

J13. Where is the visit? (can choose more than one place)

☐(01) Respondent's home ☐ (02) Respondent's office☐(03) Respondent's School ☐ (04) Other person's home☐(05)Others, please specify ____________

J14. Are there other people present when you visit?

☐(1)No (skip to K0) ☐(2)Yes

J14a. If so, who? (can choose more than one)

☐ (1) Children/son or daughter in law ☐ (2) Spouse ☐ (3) Brothers and sisters (or their spouses)☐(4)Parents/in-laws ☐(5)grandparents ☐(6) great-grandparents
☐(7) Grandchildren or his or her spouse ☐(8) Aunt/ Uncle (or his or her spouse)
☐(9) Church (table) brothers and sisters (or their spouses) ☐ (10) other relatives
☐(11) Domestic help ☐ (12) Neighbors or friends ☐ (13) Classmates or colleagues
☐(14) Others, please specify ____________

R. Supervisor records

Date of receipt of the questionnaire: ________________

R1. How many “jump-and-answer errors” in the entire questionnaire.
R2. How many questions were skipped in the entire questionnaire?
R3. How many "wrong answers" in the whole questionnaire
R4. Others, please specify: ___________ times
R5. Questionnaire status:
☐01. Complete
☐02. the interviewer must return to ask questions (continued to R5a)
☐03. the supervisor must return to ask questions (jumping R5b)
☐04. the interviewer and the supervisor must return to ask questions (continued to R5a, R5b)
R5a. The interviewer asked _______ questions and completed them after ______ days.
R5b. The interviewer asked _______ questions, it was completed after ______.
R6. Inspection date: ____ year ____ month ____ day
R7. Completion date: ____ year ____ month ____ day
R8. Review date: ____ year ____ month ____ day
R9. Review mode:
☐01. telephone
☐02. Field
☐03. Cannot find respondents
R10. Supervisor signature: ________________
R11. Supervisor number: ________________
R12. Signature of the assistant entering the data for the first time: ________________
Signature of the assistant entering the data for the second time: ________________
