# Supplementary material for: Gender differences in related influential factors of regular exercise behavior among people in Taiwan in 2007: A cross-sectional study
Source: PLoS One. 2020 Jan 31;15(1):e0228191. doi: 10.1371/journal.pone.0228191 (PMC6993962; doi:10.1371/journal.pone.0228191)
Supplement: S2 Questionaire — (PDF) [file pone.0228191.s002.pdf]

受訪者編號：________________________

訪問開始時間_____月_____日_____時_____分（二十四小時制）

A、基本狀況

A1.性別：□(01)男　□(02)女

A2.請問您是什麼時候出生的？民國_________年_________月

A3.請問您目前居住在哪裡？

_________省（市）_________縣（市）_________鄉（鎮、市、區）　郵遞區號□□□

A4.請問您覺得您住的地區是都市或鄉村？

□(01)大都市　□(02)大都市旁的郊區

□(03)小城鎮　□(04)農村地區

□(05)獨立農家

A5.請問您父親是哪裡人？

□(01)台灣閩南人　□(02)台灣客家人　□(03)大陸各省市

□(04)台灣原住民　□(05)東南亞　□(06)其他，請說明_____

A6.請問您父親的教育程度是?

□(01)無/不識字　□(02)自修　□(03)小學

□(04)國(初)中　□(05)初職　□(06)高中普通科

□(07)高中職業科　□(08)高職　□(09)士官學校

□(10)五專　□(11)二專　□(12)三專

□(13)軍警專修班　□(14)軍警專科班　□(15)空中行專

□(16)空中大學　□(17)軍警官學校

□(18)技術學院、科大　□(19)大學　□(20)碩士

□(21)博士　□(22)其他，請說明____________

A7 請問您父親在您出生後到現在有沒有工作？

□(01)有，一直有工作到現在、退休或過世

□(02)有，一直有兼職工作

□(03)有時候有，有時候沒有　□(04)沒有工作過

A8.請問您母親是哪裡人？

□(01)台灣閩南人　□(02)台灣客家人　□(03)大陸各省市

□(04)台灣原住民　□(05)東南亞　□(06)其他，請說明______

A9.請問您母親的教育程度是?

□(01)無/不識字　□(02)自修　□(03)小學

□(04)國(初)中　□(05)初職　□(06)高中普通科

□(07)高中職業科　□(08)高職　□(09)士官學校

□(10)五專　□(11)二專　□(12)三專

□(13)軍警專修班　□(14)軍警專科班

□(15)空中行專　□(16)空中大學

□(17)軍警官學校　□(18)技術學院、科大

□(19)大學　□(20)碩士

□(21)博士　□(22)其他，請說明_________

A10.請問您母親在您出生後到現在有沒有工作？

□(01)有，一直有工作到現在、退休或過世

□(02)有，一直有兼職工作

□(03)有時候有，有時候沒有　□(04)沒有工作過

A11.請問您的教育程度是?

□(01)無/不識字（跳答 A14） a1101　□(02)自修（跳答 A14） a1102

□(03)小學 a1103　□(04)國(初)中 a1104　□(05)初職 a1105

□(06)高中普通科 a1106　□(07)高中職業科 a1107　□(08)高職 a1108

□(09)士官學校 a1109　□(10)五專 a1110　□(11)二專 a1111

□(12)三專 a1112　□(13)軍警專修班 a1113　□(14)軍警專科班 a1114

□(15)空中行專 a1115　□(16)空中大學 a1116　□(17)軍警官學校 a1117

□(18)技術學院、科大 a1118　□(19)大學 a1119

□(20)碩士 a1120　□(21)博士 a1121　□(22)其他，請說明________

A12.請問您有沒有畢業？

□(01)沒有，肄業 a1201　□(02)沒有，就學中 a1202　□(03)有 a1203

A13.請問您從國小到現在，總共受過幾年的學校教育？_________年

A14.請問您目前的婚姻狀況是？

□(01)已婚有偶（跳答 A16） a1401　□(02)配偶去世 a1402

□(03)離婚 a1403　□(04)分居 a1404

□(05)單身，從未結婚 a1405　□(06)其他，請說明___________

A15 請問你目前有沒有同居伴侶？　□(01)有 a1501　□(02)沒有 a1502

A16.請問您目前有沒有信什麼教？

□(01)佛教 a1601　□(02)道教 a1602　□(03)民間信仰 a1603

□(04)一貫道 a1604　□(05)回教 a1605　□(06)天主教 a1606

□(07)基督教 a1607　□(08)沒有宗教信仰 a1608　□(09)其他，請說明______

A17.請問您常不常參加宗教活動，大概多久一次？（「無宗教信仰」者也請回答此題）

□(01)每星期好幾次　□(02)每星期一次

□(03)一個月兩三次　□(04)一個月一次

□(05)一年好幾次　□(06)一年一次

□(07)幾乎沒有（比一年一次更少）　□(08)從未參加

A18.請問您現在有沒有在工作？

□(01)有全職工作　□(02)有兼職工作

□(03)不固定（打零工），目前暫時沒有

□(04)幫忙家裡的事業

□(05)目前沒有工作（跳答 A20）

□(06)學生／進修在學且沒有工作（跳答 A20）

□(07)已經退休　□(08)家庭主婦且沒有工作（跳答 A20）

□(09)高齡、身心障礙、生病不能工作（跳答 A20）

□(10)其他，請說明______________

A19. 請問您現在做什麼工作？（若已退休，則問退休前）請詳細填寫

a.公司名稱______________________________________

主要產品、服務內容______________________________________　行業□□□

b.部門（無部門者免填）________________，職位________________變遷職位□□□

詳細工作內容____ISCO88□□□□

c.請問您平均每個星期工作幾小時？__________小時

d.請問在您的工作上，您有沒有管理其他員工？

□(01)有　□(02)沒有

e.請問您的工作性質爲何？

□(01)在政府部門工作（跳答 A20）

□(02)在公營企業工作（跳答 h）

□(03)自己開業

□(04)其他性質的工作（跳答 h）

f.請問您有沒有雇用員工？□(01)有 □(02)沒有（跳答 h）

g.請問您雇用多少員工？____________位

h.請問您現在或以前有沒有加入工會？

□(01)現在有 □(02)以前有，但現在沒有

□(03)從未加入

＜目前單身且沒有同居伴侶跳答 A22，喪偶跳答 A21＞

A20.請問您的配偶（同居伴侶）現在有沒有在工作？

□(01)有全職工作 □(02)有兼職工作

□(03)不固定（打零工），目前暫時沒有

□(04)幫忙家裡的事業 □(05)目前沒有工作（跳答 A22）

□(06)學生／進修在學且沒有工作（跳答 A22）

□(07)已經退休 □(08)家庭主婦且沒有工作（跳答 A22）

□(09)高齡、身心障礙、生病不能工作（跳答 A22）

□(10)其他，請說明____________

A21.請問您的配偶（同居伴侶）現在做什麼工作？請詳細填寫
（若已退休，則問退休前；若已過世，則問過世前）

a.公司名稱＿＿＿＿＿＿＿＿＿＿＿＿＿＿＿＿＿＿＿＿＿＿＿＿

主要產品、服務內容＿＿＿＿＿＿＿＿＿＿＿＿＿＿＿＿＿＿＿＿＿ 行業□□□

b.部門（無部門者免填）＿＿＿＿＿＿＿，職位＿＿＿＿＿＿＿＿＿＿＿變遷職位□□□

詳細工作內容＿＿＿＿＿＿＿＿＿＿＿＿＿＿＿＿＿＿＿＿＿＿＿ISCO88□□□□

c.請問您的配偶（同居伴侶）工作性質爲何？

□(01)在政府部門工作 a21c01　□(02)在公營企業工作 a21c02

□(03)自己開業 a21c03

□(04)其他性質的工作 a21c04

A22. 請問您家中，包含您本人在內，現在有幾個人住在一起? (a)＿＿＿＿＿個人，

其中有幾位是 65 歲以上（包含本人）？(b)＿＿＿位，有幾位是 6 歲以下?(c)＿＿＿位

B. 生活態度

請問下列這些說法，符不符合您的想法?<請訪員出示卡片 1>

	很符合	還算符合	無所謂符不符合	不太符合	很不符合	無法選擇
B1. 老是看到熟面孔，我會覺得很無趣	01	02	03	04	05	97
B2. 有時候我喜歡去做有點冒險的事	01	02	03	04	05	97
B3. 我想要自己一個人去陌生的地方探險	01	02	03	04	05	97
B4. 我想要跟很多人一起去陌生的地方探險	01	02	03	04	05	97

每個人對「休閒」都可能有不同的看法，您認為下面這些算不算是休閒？<請訪員出示卡片 2>

	一定算	大概算	大概不算	一定不算	無法選擇
B5. 做讓自己快樂的事	01	02	03	04	97
B6. 讓自己放鬆	01	02	03	04	97
B7. 什麼事也不做	01	02	03	04	97
B8. 單純想做，而不計回報的事	01	02	03	04	97
B9. 做自己想要做的事	01	02	03	04	97
B10. 打發(消磨)多出來的時間	01	02	03	04	97
B11. 做跟工作完全沒關係的事	01	02	03	04	97

C ISSP 2007 年題組 休閒時間: 活動與滿意程度

以下問題是有關您個人的『自由時間』，所謂自由時間是指在工作、家務以及其他必需的日常活動之外，能依自己意思做事的時間。

C1 在您的自由時間裡，您大約多久一次從事下列的活動？

	每天	一週好幾次	一月好幾次	一年好幾次或更少	從來沒有
a. 看電視、DVD、錄影帶	01	02	03	04	05
b. 到電影院看電影	01	02	03	04	05
c. 逛街購物(目的為娛樂消遣)	01	02	03	04	05
d. 看書	01	02	03	04	05
e. 參加藝文活動，例如音樂會、戲劇表演、展覽	01	02	03	04	05
f. 跟親戚聚會家族聚會 <註：指沒有一起同住的家人或親戚>	01	02	03	04	05
g. 跟朋友聚會	01	02	03	04	05
h. 玩牌或下棋	01	02	03	04	05
i. 聽音樂	01	02	03	04	05
j. 從事體能活動，例如運動，上健身房、散步	01	02	03	04	05
k. 到現場看體育比賽	01	02	03	04	05
l. 做手工藝，例如裁縫、工藝	01	02	03	04	05
m. 使用電腦或上網	01	02	03	04	05

C2 請問您在自由時間所做上面的這些事情，能不能幫你？

	幫助很大	相當有幫助	有點幫助	不太有幫助	一點幫助也沒有	無法選擇
a. 表現出真正的自己	01	02	03	04	05	97
b. 增進人際關係	01	02	03	04	05	97

C2a1.請問您在從事這些休閒活動的時候，通常是和親朋好友一起做，還是自己一個人做？

□(01)幾乎都是和親朋好友一起 □(02)大部份和親朋好友一起

□(03)大概一半一半 □(04)大部份自己一個人

□(05)幾乎都是自己一個人(跳答 C2a3)

C2a2請問您和別人在一起休閒的時候，通常是和什麼人在一起？ (最多選兩項)

□(01)配偶 □(02)父母 □(03)小孩 □(04)其他家人或親戚

□(05)同事 □(06)鄰居 □(07)朋友 □(08)其他，請說明________

C2a3最近三年來，您在平常的休閒活動中大概每次(最多)會和多少人接觸？(指一對一的接觸，包括打招呼、講話、打電話、寫信、透過電腦網路，不管認識或不認識的都算)

□(01) 0 人～4 人 c2a301　□(02) 5-19 人 c2a302

□(03) 20-99 人 c2a303　□(04) 100 人以上 c2a304

C2a4 最近三年來，您透過休閒活動認識而**成為朋友的**大概有幾位？

□(01)0 人 c2a401　□(02)1-4 人 c2a402

□(03) 5-19 人 c2a403　□(04) 20 人以上 c2a404

C2a5.請問您希望從上面這些休閒活動中交到朋友嗎？

□(01) 很希望 c2a501　□(02)有點希望 c2a502

□(03)不太希望 c2a503　□(04)很不希望(無所謂) c2a504

C3 請問您在自由時間所做下面這些活動時，享受到多少樂趣？

	完全沒樂趣	沒有什麼樂趣	有一些樂趣	相當有樂趣	非常有樂趣	從來不做
a. 看書	01	02	03	04	05	06
b. 跟朋友聚會	01	02	03	04	05	06
c. 從事體能活動，例如：運動、上健身房、散步	01	02	03	04	05	06
d. 看電視、DVD、錄影帶	01	02	03	04	05	06

時間與休閒的意義，和工作與生活其他領域的關係

C4 請問你常不常利用自由時間做下面這些事情？

	總是	經常	有時候	很少	從來沒有	無法選擇
a. 建立有用的人際關係	01	02	03	04	05	97
b. 放鬆及恢復元氣	01	02	03	04	05	97
c. 嘗試學習或增進技能	01	02	03	04	05	97

C5A.在您的自由時間裡，你常不常

	總是	經常	有時候	很少	從來沒有	不適用
a. 覺得無聊	01	02	03	04	05	99
b. 覺得匆忙	01	02	03	04	05	99
c. 發現自已在想跟工作有關的事情	01	02	03	04	05	99

C5B.在您的自由時間裡，您比較喜歡和別人在一起，還是喜歡自己一個人？

□(01)總是喜歡與別人在一起

□(02)比較喜歡跟別人在一起，勝過獨處

□(03)比較喜歡獨處，勝過跟別人在一起

□(04)總是喜歡自己一個人　□(05)無法選擇

C6.假設您可以重新安排您的時間，您希望在下面這些事情上多花時間、少花時間，還是和現在一樣？

	多花很多時間	多花一點時間	和現在一樣	少花一點時間	少花很多時間	無法選擇	不適用
a. 工作賺錢	01	02	03	04	05	97	99
b. 做家事	01	02	03	04	05	97	99
c. 陪家人	01	02	03	04	05	97	99
d. 休閒活動	01	02	03	04	05	97	99

C7a.在過去一年中，請問您大約有幾個晚上因爲度假或聚會在外面過夜？

□(01)都在家　□(02)1 到 5 個晚上

□(03)6 到 10 個晚上　□(04)11 到 20 個晚上

□(05)21 到 30 個晚上　□(06)超過 30 個晚上

□(07)無法選擇

C7b.在過去一年中，請問您大概有幾天休假沒有工作？（不包括公假、例假日、產假、育嬰假、病假或其他類似的事假）

□(01)沒有　□(02)1 到 5 天

□(03)6 到 10 天　□(04)11 到 20 天

□(05)21 到 30 天　□(06)超過 30 天

□(07)無法選擇　□(08)沒有在工作

運動/遊戲活動及其主觀功能

C8a.請問您最常做的運動或體能活動是什麼？＿＿＿＿＿＿＿＿＿　代碼□□□

□(01)我不從事任何運動或體能活動（跳答 C8b）

C8a1.請問您大概多久沒做這項運動或體能活動，就會有不太舒服或不太對勁的感覺？

□(1)一天 c8a101　□(2)兩到三天 c8a102

□(3)一個禮拜左右 c8a103　□(4)兩個禮拜左右 c8a104

□(5)一個月左右或更久 c8a105　□(6)從來沒有 c8a106

C8a2.請問您第二常做的運動或體能活動是什麼＿＿＿＿＿＿＿＿＿＿　代碼□□□

□(01)我沒有第二常做的運動 c8a201

C8b.在運動或體能活動之外，您最常玩什麼遊戲？（單選題）＜請訪員出示卡片 3＞

紙板遊戲

□01 西洋雙陸棋戲 c8b01

□02 西洋跳棋 c8b02

□03 西洋棋 c8b03

□04 圍棋 c8b04

□05 其他紙板遊戲（例如：大富翁、英文拼字遊戲） c8b05

□06 紙牌遊戲（例如：橋牌、鬼佬麻雀、單人牌戲等） c8b06

□07 骨牌 c8b07

□08 麻將 c8b08

□09 拼圖 c8b09

□10 文字或數字遊戲（例如：縱橫填字謎、數獨遊戲） c8b10

□11 電動遊戲、電腦遊戲、PS 電視遊樂器、彈鋼珠遊戲 c8b11

□12 賭博遊戲（例如：賭場遊戲、吃角子老虎、樂透、體育賭博） c8b12

□13 各國的特殊遊戲（象棋） c8b13

□14 其他遊戲： c8b14

□15 我不玩任何遊戲 c8b15

C9. 對您而言，下述參與運動或遊戲的理由是否重要？

	很重要	有點重要	不太重要	不重要	無法選擇	不適用
a. 為了身心健康	01	02	03	04	97	99
b. 為了跟別人接觸	01	02	03	04	97	99
c. 為了與他人競賽	01	02	03	04	97	99
d. 為了讓身材好看	01	02	03	04	97	99
e. 為了興趣	01	02	03	04	97	99

C10a 請問您最常看哪一類的電視體育節目或運動比賽？________________ 代碼□□□

□(01)我不看任何電視體育運動節目（跳答 C11） c10a01

C10b 請問您第二常看的電視體育節目或運動比賽？______________ 代碼□□□

□(01)我沒有第二常看電視體育運動節目 c10b01

運動與休閒的社會學觀點

C11. 當台灣的選手在國際運動或其他競技比賽中表現優異時，您覺得光不光榮？

□(01)我覺得很光榮 c1101　□(02)我覺得有點光榮 c1102

□(03)我並不覺得很光榮 c1103　□(04)我一點都不覺得光榮 c1104

□(05)無法選擇 c1105

C12. 每個人對於運動有不同的看法，請問您是否同意下列的說法？

	非常同意	同意	不同意也不反對	不同意	非常不同意	無法選擇
a. 參加運動對孩童的個性發展有幫助	01	02	03	04	05	97
b. 電視上的運動節目太多	01	02	03	04	05	97
c. 運動拉近了國內不同團體及種族的距離	01	02	03	04	05	97
d. 國際運動競賽比較容易造成國家間關係緊張，不容易培養良好的氣氛	01	02	03	04	05	97
e. 政府應該編列更多的經費在運動項目上	01	02	03	04	05	97

社會與政治參與

C13 在過去一年來，您常不常參加下面這些團體的活動？

	一週至少一次	一個月至少一次	好幾次	一兩次	從來沒有
1. 體育運動協會/團體	01	02	03	04	05
2. 文化協會/團體	01	02	03	04	05
3. 教會或其他宗教組織	01	02	03	04	05
4. 社區服務或一般民間協會/團體	01	02	03	04	05
5. 政黨或政治組織	01	02	03	04	05

C14a.你認爲跟人來往時，一般人大都可以信任，還是對人必須非常小心？

□(01)總是可以信任　□(02)通常可以信任

□(03)通常必須小心　□(04)總是必須小心

□(05)無法選擇

C14b 請問您個人對政治有多大的興趣？

□(01)很有興趣　□(02)還算有興趣

□(03)不太有興趣　□(04)一點也沒興趣

□(05)無法選擇

休閒的社會決定因素和後果

C15.以下條件對你在『自由時間』所從事的活動影響有多大？

	非常大	很大	有一點	完全沒有	無法選擇
a. 附近沒有設施	01	02	03	04	97
b. 沒有錢	01	02	03	04	97
c. 個人的健康狀況（年紀、行動不便）	01	02	03	04	97
d. 需要照顧其他人（老人或小孩）	01	02	03	04	97
e. 沒有時間	01	02	03	04	97

C16 整體而言，您覺得您的生活快樂還是不快樂？

□(01)非常快樂 □(02)還算快樂
□(03)不太快樂 □(04)一點都不快樂
□(05)無法選擇

C17. 一般而言，您認爲您的健康情況如何？

□(01)非常好 □(02)很好
□(03)好 □(04)還可以
□(05)差 □(06)無法選擇

D 運動

D1 請問您贊成您的子女成為職業運動員嗎？（沒有子女也要問）

□(01)很贊成 □(02)還算贊成
□(03)不太贊成 □(04)很不贊成

D2.請問您認為下列那種說法比較適合用來描述『拳擊』這項運動？

□(01)靠力量但也有技巧的運動 □(02)粗暴野蠻的運動
□(03)無意見 □(04)其它，請說明_____

D3 請問您認為下列那種說法比較適合用來描述『高爾夫球』這項運動？

□(01)高尚的運動 □(02)昂貴的運動
□(03)無意見 □(04)其它，請說明__________

D4 請問您認為在籃球比賽中肢體碰撞是？

□(01)合理的競技策略 □(02)無法避免的正常現象
□(03)應避免的粗魯行為 □(04)無意見
□(05)其它，請說明______

D5 如果運動比賽中對手故意撞人，請問您認為可不可以撞回去？

□(01)一定可以 d501 □(02)大概可以 d502

□(03)大概不可以 d503 □(04)一定不可以 d504

□(97)不知道 d597

D6 請問您認為在運動比賽中向對手叫罵（台語：嗆聲）算是合理的心理戰略運用嗎？

□(01)一定算 d601 □(02)大概算 d602

□(03)大概不算 d603 □(04)一定不算 d604

□(97)不知道 d697

D7 請問您認為值不值得花錢做運動？

□(01)非常值得 d701 □(02)還算值得 d702

□(03)不太值得 d703 □(04)非常不值得 d704

D8 請問您同不同意，運動只要有動就好，不必太要求自己

□(01)非常同意 d801 □(02)同意 d802

□(03)不同意也不反對 d803 □(04)不同意 d804

□(05)非常不同意 d805

D9 請問您認為下列幾項活動算是運動嗎？

	一定算	大概算	大概不算	一定不算	無意見	不知道
a.太極拳	01	02	03	04	05	97
b.氣功	01	02	03	04	05	97
c.宋江陣或八家將	01	02	03	04	05	97
d.瑜珈	01	02	03	04	05	97

D10 在您認定某項活動是或不是運動時，下列這些條件重不重要？<請訪員出示卡片 4>

	非常重要	有點重要	不太重要	不重要	無意見	不知道
a.有競賽的性質	01	02	03	04	05	97
b.有明確的規則	01	02	03	04	05	97
c.身體活動量夠大	01	02	03	04	05	97
d.能夠養生	01	02	03	04	05	97
e.能夠健身	01	02	03	04	05	97

E 觀光

E1 請問你喜不喜歡下面這些觀光旅行的型態？＜請訪員出示卡片 5＞

	非常喜歡	很喜歡	有點喜歡	不太喜歡	不知道	拒答
a. 參加戶外冒險（例如泛舟、滑雪、攀岩）	01	02	03	04	97	98
b 欣賞歷史文物（例如博物館、美術館）	01	02	03	04	97	98
c 享受定點度假（例如度假村、休閒飯店）	01	02	03	04	97	98
d 觀賞自然風景（例如大峽谷、瀑布）	01	02	03	04	97	98
e.逛街購物（例如百貨公司、名牌精品）	01	02	03	04	97	98

E2 請問您**最主要**是如何安排您的**國內**旅遊行程：＜請訪員出示卡片 6＞

☐(01)從來沒參加過（跳答 E4）　e201

☐(02)參加親友、公司、社團、社區的集體旅遊　e202

☐(03)向旅行社報名參加觀光團（跟團）　e203

☐(04)自由行、半自助旅行、自助旅行　e204

☐(05)其他，請說明＿＿＿＿＿＿＿

E3 **在過去三年中**，您在**國內**觀光旅行幾次？＿＿＿次

E4 你最近想不想在國內觀光旅行 ？

☐(01)非常想　e401　☐(02)有點想　e402

☐(03)不太想　e403　☐(04)完全不想　e404

☐(97)不知道　e497　☐(98)拒答　e498

E5 您**最主要**是如何安排您的**國外**旅遊行程：＜請訪員出示卡片 6＞

☐(01)從來沒參加過（跳答 E7）　e501

☐(02)參加親友、公司、社團、社區的集體旅遊　e502

☐(03)向旅行社報名參加觀光團（跟團）　e503

☐(04)自由行、半自助旅行、自助旅行　e504

☐(05)其他，請說明 ＿＿＿＿＿＿＿＿

E6 **在過去三年中**，您到**國外**觀光旅行幾次？______ 次

E7 你最近想不想到國外觀光旅行？

□(01)非常想 □(02)有點想

□(03)不太想 □(04)完全不想

□(97)不知道 □(98)拒答

E8 工作和休閒，您覺得哪個比較重要？

□（01）工作 □（02）休閒

E9 想一下在您主要的工作中，包含經常性加班的工作時數與收入在內您會希望：（單選題）

□(01)為了多賺一點錢，工作時間久一點

□(02)工作時間一樣，賺同樣的錢

□(03)工作時間短一點、少賺一點也沒關係

□(97)不知道，無法選擇

E10 請問您可不可以自己決定怎麼安排日常的工作？

□(01)可以自由決定 □(02)多多少少可以決定

□(03)不能自由決定 □(04)無法選擇

F 休閒之紛爭解決與安全

F1 請問您或您的家人在最近 10 年內（自民國 86 年/西元 1997 年開始）從事休閒活動時，有沒有和業者發生過糾紛或不愉快？

□(01)有重大糾紛 □(02)有輕微糾紛

□(03)有小小的不愉快 □(04)沒有

F2 如果休閒活動發生糾紛造成您財產上的重大損失，但業者又不願意賠償時，請問您認為下列哪一個對象最能幫助您求償？＜請訪員出示卡片 7＞

□(01)法院 f201
□(02)檢察官 f202
□(03)律師 f203
□(04)鄉(鎮、市、區）調解委員會 f204
□(05)政府的消費者保護委員會（消保會） f205
□(06)民間的消費者文教基金會（消基會） f206
□(07)業者投保的保險公司 f207
□(08)您（消費者本身）投保的保險公司 f208
□(09)民意代表 f209
□(10)新聞媒體 f210
□(11)其他，請說明________

F3 接續前一題，如果造成您身體上的重大傷害時，請問您認為下列哪一個對象最能幫助您求償？＜請訪員出示卡片 7＞

□(01)法院 f301
□(02)檢察官 f302
□(03)律師 f303
□(04)鄉(鎮、市、區）調解委員會 f304
□(05)政府的消費者保護委員會（消保會） f305
□(06)民間的消費者文教基金會（消基會） f306
□(07)業者投保的保險公司 f307
□(08)您（消費者本身）投保的保險公司 f308
□(09)民意代表 f309
□(10)新聞媒體 f310
□(11)其他，請說明_______

F4 如果在民營的休閒活動場所發生天災、地震等意外事故，造成消費者財產上的重大損失，但業者沒有過失，您認為誰應該負主要的賠償責任？
＜請訪員出示卡片 8＞

□(01)消費者自己承擔 f401
□(02)業者負責 f402
□(03)政府負責 f403
□(04)政府跟業者都要負責 f404
□(05)業者投保的保險公司 f405
□(06)您（消費者本身）投保的保險公司 f406

F5 接續前一題，若是有人因此受傷或死亡時，您認為誰應該負主要的賠償責任？

＜請訪員出示卡片 8＞

□(01)消費者自己承擔	f501	□(02)業者負責	f502
□(03)政府負責	f503	□(04)政府跟業者都要負責	f504
□(05)業者投保的保險公司	f505	□(06)您（消費者本身）投保的保險公司	f506

F6 請問您認為國家應不應該立法規定休閒娛樂場所，都要有特別提供給行動不便者的服務或措施，否則業者就要受罰？

□(01)絕對有必要	f601	□(02)應該有必要	f602
□(03)不太有必要	f603	□(04)沒必要	f604

F7 一般而言，游泳池的月票或年票收費男女價錢是一樣的。如果因為女性的生理期因素，而給女性較便宜的票價或者較長的使用期限，您認為這樣合不合理？

□(01)很合理	f701	□(02)還算合理	f702
□(03)不太合理	f703	□(04)很不合理	f704

F8 休閒場所的置物櫃上標示「請勿放置貴重物品，如有遺失不負賠償責任」。請問您認為這對消費者而言合不合理？

□(01)很合理	f801	□(02)還算合理	f802
□(03)不太合理	f803	□(04)很不合理	f804

F9 請問您到電影院或 KTV 時，會不會檢查或注意它的消防設施是否安全？

□(01)一定會	f901	□(02)有時候會	f902
□(03)很少會	f903	□(04)一定不會	f904
□(99)不適用/沒去過	f999		

F10 請問您認為政府會不會定期檢查『電影院或 KTV』的消防設施？

□(01)一定會 f1001　□(02)大概會 f1002

□(03)大概不會 f1003　□(04)一定不會 f1004

F11 請問您認為政府有沒有確實檢查『電影院或 KTV』的消防設施？

□(01)一定有 f1101　□(02)有時候有 f1102

□(03)很少有 f1103　□(04)一定沒有 f1104

F12 請問您認為『遊樂場業者』會不會定期檢查維護『遊樂設施』的安全性？

□(01)一定會 f1201　□(02)大概會 f1202

□(03)大概不會 f1203　□(04)一定不會 f1204

F13 請問您認為『遊樂場業者』有沒有確實檢查維護『遊樂設施』的安全性？

□(01)一定有 f1301　□(02)有時候有 f1302

□(03)很少有 f1303　□(04)一定沒有 f1304

F14 請問您到休閒農場或民宿時，會不會檢查或注意它有無政府認證的合格書？

□(01)一定會 f1401　□(02)有時候會 f1402

□(03)很少會 f1403　□(04)一定不會 f1404

□(99)不適用/沒去過 f1499

F15 請問您參加旅行社的旅行團時，您本人會不會先親自閱讀除了個人資料及行程以外的契約內容？ ＜請訪員出示卡片 9＞

□(01)會，仔細閱讀 f1501　□(02)會，大概看一遍 f1502

□(03)不會，只向旅行社人員口頭詢問 f1503　□(04)不會，直接聽從旅行社人員的指示簽名 f1504

□(05)不會，都是交給親友或主辦單位決定 f1505

F16 請問您認為和旅行社簽訂的契約，在糾紛發生時可不可以有效保障您的權益？

□(01)一定可以 f1601　□(02)大概可以 f1602

□(03)大概不可以 f1603　□(04)一定不可以 f1604

□(98)拒答 f1698

F17 一般旅行社於出團前會說已經依法令投保了旅遊業綜合責任保險，您知不知道主要是為了誰的利益而投保？<請訪員出示卡片 10>

□(01)不知道有投保 f1701　□(02)知道，是團員 f1702

□(03)知道，是旅遊社 f1703　□(04)知道，是團員與旅遊社 f1704

□(05)不知道是為誰的利益投保 f1705

G 休閒閱讀題組

G1 請問你最近三年平均每個星期看書大概看多久？(不含報紙及雜誌) ______小時______分鐘

□(1)從來不看書（跳答 H1） g101

G2 在你的自由時間裡，請問你是否常閱讀下面這幾種書？

	經常	有時	很少	從來沒有
a.愛情文藝小說	01	02	03	04
b.武俠、偵探、奇幻等小說	01	02	03	04
c.家庭生活(DIY、健康、教育）	01	02	03	04
d.影視娛樂/時尚流行	01	02	03	04
e.藝術文化/觀光旅遊	01	02	03	04
f.消費理財/財經產業	01	02	03	04
g.科學/資訊科技	01	02	03	04
h.心靈勵志/宗教	01	02	03	04
i.古典文學/現代文學	01	02	03	04
j.人物傳記/歷史	01	02	03	04
k.暢銷書	01	02	03	04
l.漫畫	01	02	03	04
m.其他，請說明____________	01	02	03	04

G3 根據您看書的經驗，符不符合下列說法？

	非常符合	很符合	還算符合	不太符合
a.看書帶給我生活樂趣	01	02	03	04
b.看書幫助我增加知識	01	02	03	04
c.看書讓我和別人互動時增加話題	01	02	03	04
d.看書幫助我放鬆心情	01	02	03	04

H.個性與生活感受

H1您碰到放假的時候，常不常煩惱要怎麼打發時間或安排出去玩？

□(01)經常　　□(02)有時候

□(03)很少　　□(04)幾乎沒有

H2 請問您過去一個月以來的休閒活動常不常讓您有下面這些感受?

	經常	有時候	很少	幾乎沒有
a.我的休閒活動對我來說很有趣	01	02	03	04
b.我的休閒活動增加了我對身邊事物的知識	01	02	03	04
c.我可以透過休閒活動和別人互相來往	01	02	03	04
d.我的休閒活動幫助我放鬆	01	02	03	04
e.我的休閒活動幫助我維持健康	01	02	03	04
f.我從事休閒活動的地方設計得不錯	01	02	03	04

H3 您認為下面這些特徵符不符合您自己的個性？

	很符合	有點符合	不太符合	很不符合
a 不太愛說話	01	02	03	04
b 很有同情心	01	02	03	04
c 事情做到完為止	01	02	03	04
d 懂得放鬆而且會處理壓力	01	02	03	04
e 想像力豐富	01	02	03	04
f 外向、會和人交際	01	02	03	04
g 不太信任別人	01	02	03	04
h 粗心大意	01	02	03	04
i 容易緊張	01	02	03	04
j 是保守的人	01	02	03	04

H4 請問您有沒有下面這種經驗或感覺？

	經常	有時候	很少	從來沒有
a 想要佔人家便宜	01	02	03	04
b 嫉妒人家運氣好	01	02	03	04
c 講別人閒話	01	02	03	04

H5 請問您平常一天裏面，大概總共跟多少人接觸？《指一對一的接觸，包括點頭、打招呼、講話、打電話、寫信、透過電腦網路，不論認識或不認識的，都算在一起》

□(01)0-4 人　□(02) 5-9 人　□(03)10-19 人

□(04)20-49 人　□(05)50-99 人　□(06)100 人以上

H6 請問您平常聽音樂時，最喜歡聽那一種類型的音樂？(最多選三項)＜請訪員出示卡片 11＞

□(01)不聽音樂　□(02)歌仔戲

□(03)國劇（或其它地方戲曲）　□(04) 國樂

□(05)台語老歌　□(06)台語流行歌曲

□(07)國語老歌　□(08)國語流行歌曲

□(09)西洋老歌　□(10)西洋流行歌曲

□(11)日語流行歌曲　□(12)韓語流行歌曲

□(13)古曲音樂（含歌劇）　□(14)藍調或爵士

□(15)搖滾或嘻哈　□(16)音樂劇

□(17)其它，請說明______

H7 請問您最喜歡吃那一種類型的料理？(最多選三項)＜請訪員出示卡片 12＞

□(01)台菜　□(02)大陸各省的料理（如川菜、江浙菜）

□(03)客家菜　□(04)美式料理（如牛排、漢堡）

□(05)歐式料理（如法國菜、德國菜、義大利菜）　□(06)日本料理

□(07)東南亞料理（如泰國菜、越南菜）　□(08)其它，請說明______

H8 請問您平常外出用餐時，最喜歡到哪裡用餐？

□(01)路邊攤 h801　□(02)小吃店 h802

□(03)百貨公司或商場的美食街 h803　□(04)有特色的餐館或高級餐廳 h804

□(05)其他，請說明_____

H9 你平常一個星期大概有幾天在外面吃晚餐？ _________天

H10 請問您平常大約幾點起床? _______點 ______分　（二十四小時制）

H11 請問您平常大約幾點睡覺? _______點 ______分　（二十四小時制）

H12 當警方抓到刑事案件的嫌犯，您認為被害人（或其家屬）動手打嫌犯有沒有道理？

□(01)非常有理 h1201　□(02)還算有理 h1202

□(03)不太有理 h1203　□(04)非常沒理 h1204

□(05)無意見 h1205

H13 請問您認為找黑道幫忙討債會不會比打官司還有效？

□(01)一定會 h1301　□(02)大概會 h1302

□(03)大概不會 h1303　□(04)一定不會 h1304

□(97)不知道 h1397

I. 收入狀況

I1. 目前國內有幾個主要政黨，包括國民黨、民進黨、親民黨、新黨以及台灣團結聯盟，請問您有沒有比較偏向那一個政黨？

□(01)國民黨 i101　□(02)民進黨 i102　□(03)新黨 i103

□(04)親民黨 i104　□(05)台聯 i105　□(06)無黨籍 i106

□(08)沒有偏向哪一黨 i108　□(07)其他，請說明______

I2. 如果整個社會由上而下分為十層，第一層代表社會地位最低，第十層代表最高。請問您認為自己的社會地位屬於第＿＿＿＿＿＿層

I3. 請問您個人平均每個月所有的(稅前)收入差不多有多少？（包括薪資、年終獎金、年節分紅、加班費、執行業務收入、自營收入、投資利息、房租、退休金、或父母/小孩給予的生活費等收入）

□(01)無收入	i301	□(02)1 萬元以下	i302
□(03)1-2 萬元以下	i303	□(04)2-3 萬元以下	i304
□(05)3-4 萬元以下	i305	□(06)4-5 萬元以下	i306
□(07)5-6 萬元以下	i307	□(08)6-7 萬元以下	i308
□(09)7-8 萬元以下	i309	□(10)8-9 萬元以下	i310
□(11)9-10 萬元以下	i311	□(12)10-11 萬元以下	i312
□(13)11-12 萬元以下	i313	□(14)12-13 萬元以下	i314
□(15)13-14 萬元以下	i315	□(16)14-15 萬元以下	i316
□(17)15-16 萬元以下	i317	□(18)16-17 萬元以下	i318
□(19)17-18 萬元以下	i319	□(20)18-19 萬元以下	i320
□(21)19-20 萬元以下	i321	□(22)20-30 萬元以下	i322
□(23)30 萬元以上	i323		

I4. 包括各種收入來源，請問您全家人的所有稅前收入，每個月大約有多少（含工作收入、兼業收入與獎金、投資利息收入、紅利或股息、政府津貼、房租收入或其他收入、退休金等）？

□(01)無收入 i401
□(02)1 萬元以下 i402
□(03)1-2 萬元以下 i403
□(04)2-3 萬元以下 i404
□(05)3-4 萬元以下 i405
□(06)4-5 萬元以下 i406
□(07)5-6 萬元以下 i407
□(08)6-7 萬元以下 i408
□(09)7-8 萬元以下 i409
□(10)8-9 萬元以下 i410
□(11)9-10 萬元以下 i411
□(12)10-11 萬元以下 i412
□(13)11-12 萬元以下 i413
□(14)12-13 萬元以下 i414
□(15)13-14 萬元以下 i415
□(16)14-15 萬元以下 i416
□(17)15-16 萬元以下 i417
□(18)16-17 萬元以下 i418
□(19)17-18 萬元以下 i419
□(20)18-19 萬元以下 i420
□(21)19-20 萬元以下 i421
□(22)20-30 萬元以下 i422
□(23)30-40 萬元以下 i423
□(24)40-50 萬元以下 i424
□(25)50-100 萬元以下 i425
□(26)100 萬元以上 i426

訪問結束時間_____月_____日_____時_____分（二十四小時制）

受訪者聯絡電話：（　）________________________;手機________________________

J 訪問記錄

J1. 訪員編號：____________

J2. 在正式訪問時，是否一次就完成訪問？

□(1)是 j201

□(2)否（請續填下列訪問開始與結束時間） j202

第一次開始時間：____月____日____點____分；結束時間：____點____分
第二次開始時間：____月____日____點____分；結束時間：____點____分
第三次開始時間：____月____日____點____分；結束時間：____點____分
（24 小時制）

J3. 在正式訪問時，這份問卷是：

□(01)受訪者自填 j301

□(02)訪員訪填，但沒有給受訪者看問卷題目 j302

□(03)訪員訪填，但有給受訪者看問卷題目 j303

□(04)透過第三者翻譯訪談 j304

□(05)其他，請說明________________

J4. 在訪問中，受訪者有沒有表示過拒絕受訪的意思？

□(01)開始時有意要拒絕 j401

□(02)訪問進行中曾表示拒絕的意思 j402

□(03)訪問到最後有拒絕的意思 j403

□(04)從頭到尾曾數次表達拒訪的意思 j404

□(05)從頭到尾均未表示拒絕 j405

J5. 在訪問中，受訪者是否表示不耐煩？

□(01)從未表示不耐煩 j501
□(02)偶爾表示不耐煩 j502
□(03) 有時不耐煩 j503
□(04) 一直不耐煩 j504

J6. 在訪問中，受訪者對訪員的信任程度如何？

□(01)很低 j601
□(02)低 j602
□(03)高 j603
□(04)很高 j604

J7. 在訪問中，受訪者是否有意應付？

□(01)大都在應付 j701
□(02)有些時候在應付 j702
□(03)不像是在應付 j703
□(04)完全沒有應付的意思 j704

J8. 受訪者合作程度：

□(01)很合作 j801
□(02)合作 j802
□(03)不合作 j803
□(04)很不合作 j804

J9. 訪問所回答的可靠程度：

□(01) 很可靠 j901
□(02)可靠 j902
□(03) 不可靠 j903
□(04)很不可靠 j904

J10. 訪問時所用的語言是：

□(01)國語 j1001
□(02)台語 j1002
□(03)客語 j1003
□(04)國台語 j1004
□(05)國客語 j1005
□(06)其他，請說明 ________

J11. 訪問時是否單獨作業？ □(1)是 □(2)否

J12. 訪問前是否有事先約定時間？ □(1)是 □(2)否

J13. 訪問是在哪裡訪問的？ （可複選）

□(01)受訪者家裡 □(02)受訪者工作處
□(03)受訪者學校 □(04)其他人家裡
□(05)其他，請說明____________

J14. 您訪問時有其他人在場嗎？

□(1)沒有(跳答 K0) □(2)有

J14a. 若有，是誰？（可複選）

□(1)子女/婿媳 □(2)配偶 □(3)兄弟姊妹(或其配偶)
□(4)父母/公婆/岳父母 □(5)祖父母/外祖父母 □(6)曾祖父母/外曾祖父母
□(7)孫兒/外孫(或其配偶) □(8)伯叔姑/舅姨(或其配偶)
□(9)堂(表)兄弟姊妹(或其配偶) □(10)其他親戚
□(11)幫傭 □(12)鄰居或朋友 □(13)同學或同事
□(14)其他人，請說明____________

R.督導記錄

收到問卷日期：民國____年____月____日
　說明：1.統計單位為「次數」，非題目
　　　　2.統計之題目包含封閉與開放型題目
　　　　3.依據統計結果之次數填答，無為 0

R1.整份問卷中「跳答錯誤」情形共有　__________次
R2.整份問卷中「題目漏問或需補問」共有　__________次
R3.整份問卷中「答案歸錯」共有　__________次
R4.其他，請說明：______________　__________次

R5.問卷狀況：□01.完整 r501
□02.退回訪員補問（續問 R5a） r502
□03.由督導補問（跳問 R5b） r503
□04.由訪員與督導補問（續問 R5a、R5b） r504

R5a.經訪員補問_______題，經過______天才完成。

R5b.經督導補問_______題，經過______天才完成。

R6.檢查日期：____年____月____日
R7.補問完成日期：____年____月____日
R8.複查日期：____年____月____日

R9.複查方式【可複選】：□01.電訪 r901
□02.實地 r902
□03.找不到受訪者 r903

R10.督導簽名：_______________

R11.督導編號：_______________

R12.計畫人員第一次輸入簽名：_______________

第二次輸入簽名：_______________
